# A Multi-Scenario Coupled Simulation of Diet–Land Systems: Diet–Land Supply–Demand Matching and Responses from the Historical-to-Future

**DOI:** 10.3390/foods15091490

**Published:** 2026-04-24

**Authors:** Liu Zhang, Xuanyun Zhang, Jiabao Zhang, Bin Fang, Chunhua Xia, Yun Ling, Kaili Zhang, Shihan Zhang, Zongchen Zhao, Xueying Lv

**Affiliations:** 1 College of Instrumentation & Electrical Engineering, Jilin University, Changchun 130061, China; zhangliu@jlu.edu.cn (L.Z.); z386372140@163.com (X.Z.); zzc1234567890323@163.com (Z.Z.); lvxueying@jlu.edu.cn (X.L.); 2 College of Geography, Nanjing Normal University, Nanjing 210023, China; xia-pikachu@foxmail.com (C.X.); 241301009@njnu.edu.cn (Y.L.); zklnwu413@163.com (K.Z.); q137003398@163.com (S.Z.)

**Keywords:** dietary structure upgrading, diet–land structure adjustment, multi-scenario coupling simulation, all-encompassing approach to food

## Abstract

Dietary transition is reshaping cropland demand and intensifying the challenge of matching food demand with land supply in rapidly urbanizing regions. This study examines how different dietary structure scenarios generate differentiated cropland demand, how these demands match with land supply under alternative development pathways, and how the land system responds when diet-driven demand is incorporated into land-use simulation. Using Jiangsu Province, China, as a case study, we developed a coupled diet–land simulation framework. On the demand side, five dietary structure scenarios—current, balanced, U.S., Japanese, and Greek—were constructed based on seven food categories, and their cropland demand in 2035 and 2050 was estimated using the cropland footprint approach and LSTM forecasting. On the supply side, the GeoSOS-FLUS model was used to simulate future land-use patterns under four development scenarios: natural development, cultivated land protection, ecological protection, and economic development. The cropland demand associated with each dietary scenario was then introduced into the land-use simulation process as an external demand constraint to identify land-system feedbacks and scenario differences. The results show that cropland demand differs markedly across dietary scenarios, forming a clear gradient from moderate-demand to high-demand diets. These differences are driven primarily by changes in the composition of key food categories, especially grains, livestock and poultry meat, plant oils, and fruits, rather than by proportional increases across all foods. In terms of supply–demand matching, the cultivated land protection scenario provides the strongest support for high-demand diets, whereas the natural development, ecological protection, and economic development scenarios are more compatible with moderate-demand dietary pathways. Once diet-driven demand is incorporated into land-use simulation, the land system shows clear sensitivity and strong scenario dependence. High-demand dietary scenarios intensify cropland compensation pressure and trigger structural reallocation among cultivated land and flexible land types. Under natural development, the response is mainly reflected in cropland expansion and grassland compression; under cultivated land protection and ecological protection, it is expressed more through substitutions among grassland, water bodies, and unused land; under economic development, the most prominent feedback is the competitive reallocation among cultivated land, construction land, and water bodies, with high dietary demand even constraining construction land expansion. Overall, the robustness of cropland supply–demand matching depends not only on the scale of dietary demand but also on how different dietary pathways interact with development-oriented land-use structures.

## 1. Introduction

Cultivated land is a fundamental resource for human survival and development, and an essential material basis for food security, food supply, and regional sustainability [1,2]. However, in the context of rapid urbanization and increasingly stringent land-use regulation, the loss of rural production land, especially cultivated land, is no longer merely a matter of quantitative decline; rather, it has evolved into a broader challenge involving the compression of agricultural production space, the weakening of food supply capacity, and growing imbalance in land-use structure [3]. Therefore, whether cultivated land loss constitutes a substantive risk depends not simply on how much land is lost, but on whether such loss undermines a region’s capacity to secure a basic food supply, respond to external shocks, and sustain long-term development [4]. Within the framework of the United Nations 2030 Agenda for Sustainable Development, SDG 2, SDG 12, and SDG 15 place higher demands on the transformation of agri-food systems from the perspectives of food security and improved nutrition, resource efficiency and sustainable consumption, and the protection of terrestrial ecosystems [5]. This implies that current cultivated land issues concern not only whether there is sufficient land for production, but also how to reconcile rising population and expanding food demand with resource conservation and ecological protection [6]. Against this background, China has introduced the all-encompassing approach to food, a policy concept that moves beyond the conventional emphasis on staple-grain security and advocates a diversified food supply system based on the coordinated development of agriculture, forestry, animal husbandry, and fisheries from a broader territorial resource perspective [7]. Meanwhile, food consumption in China has gradually shifted from a grain-centered pattern toward a more nutrition-oriented and diversified structure, with continuously increasing demand for vegetables, fruits, oil crops, feed, and other agricultural products, thereby reshaping agricultural production structures and the corresponding allocation of land resources [8]. At the same time, food security is no longer solely a matter of production, but increasingly a comprehensive issue encompassing production, distribution, and consumption [9]. In recent years, natural disasters, public health emergencies, and trade conflicts have significantly disrupted telecoupled food systems, exposing practical vulnerabilities in some regions, including insufficient local supply capacity, high external dependence, and fragile cross-regional coordination [9]. This suggests that even when food availability appears adequate at a larger scale, different regions may still face unequal risks in access to food and nutrition due to disparities in production capacity, circulation constraints, or mismatches in supply structure [10]. Furthermore, the emphasis on sustainable consumption under SDG 12 highlights that dietary upgrading should not be equated with unlimited consumption expansion, but should instead involve a transition toward healthier and more sustainable consumption under conditions of improved resource-use efficiency, reduced food loss and waste, and respect for ecological boundaries [11]. In this context, China’s recent emphasis on maintaining a stable area of permanent vegetable land in large- and medium-sized cities and on building a diversified food supply system reflects a systematic response to local food-system resilience, regional supply security, and the transition toward sustainable consumption [12]. Therefore, examining how dietary change under the all-encompassing approach to food translates into pressure on cultivated land demand, and how such pressure further affects land-use restructuring under different development goals, is of considerable theoretical and practical significance [13].

Existing research on the above issues tends to be fragmented. One type of study focuses on calculating the cultivated land demand for different foods based on consumption structures, revealing the resource pressure differences between various dietary patterns [14]. In this research paradigm, land supply is typically treated as fixed, meaning that cultivated land supply is assumed to remain stable while changes in demand are taken as the primary focus [15]. Another type of study focuses on multi-scenario land-use simulations (such as projecting future regional construction land expansion and changes in cultivated land/ecological patterns under constraints like natural development, cultivated land protection, ecological protection, and urban economic development) [16]. This type of study can effectively model spatial pattern evolution, but it often substitutes dietary structure with land-use scale targets [17]. Additionally, they tend to overlook the changes in land-use spatial patterns caused by dietary structure upgrades.

Therefore, the dynamic coupling among dietary restructuring, the upgrading of cropland demand, land-use supply capacity, and feedbacks within the food–land system remains insufficiently understood, and this gap is particularly pronounced in highly developed regions such as Jiangsu Province, China, where rapid urbanization coincides with intense land scarcity [18]. Existing studies suggest that the essence of the supply–demand relationship in food–cropland systems lies in the multidimensional negotiation between the demands of socioeconomic systems and the supply capacities of cropland resource systems across time and space [19]. Much of the existing literature draws on the theory and methods of ecosystem service supply–demand matching and uses the productive function of cultivated land as the bridge for characterizing these relationships [20]. Research has tended to focus on evaluating the carrying capacity of water and cropland resources for staple foods, livestock products, or a limited number of food categories, on macro-level analyses of national food supply–demand balances, or on equilibrium states under particular levels of food consumption [13]. Supply–demand mismatches in food–cropland systems essentially represent a spatial dislocation between cropland resource endowments and population and socioeconomic development. The consequences of such mismatches are that social demand for agricultural products may remain unmet or, conversely, product surpluses may induce inefficient or excessive cropland use, thereby generating varying degrees of potential risk for food security, social stability, and economic development [10]. One important reason is that cropland supply tends to respond more slowly than increasingly diversified human demand. When new demand emerges, managers need to adjust land-use and management in light of observed mismatches, and to re-plan and reallocate cultivated land so that supply can better meet diversified consumer needs [13]. Overall, the supply–demand matching relationship in food–cropland systems evolves dynamically alongside changes in land structure and economic transformation, while recent evidence indicates that demand-side pressures associated with dietary upgrading, urbanization, and structural change are becoming more influential than supply-side adjustments [10]. Current regulation, however, still largely remains at the level of macro-scale assessment and zoning guidance, and lacks an analytical framework that explicitly incorporates demand upgrading into the dynamic coupling analysis of land-structure change and structural reallocation. As a result, the efficiency of supply–demand coordination remains limited, and optimized pathways are difficult to translate into concrete governance actions [21].

From an environmental perspective, one study has shown that healthier dietary patterns, such as reduced meat consumption and more plant-based diets, can lower water use, land demand, and carbon emissions, while also easing pressure on land-use systems [22]. Different dietary structures place different levels of demand on land systems [23]. Dietary patterns with relatively lower land demand are generally more compatible with regional development goals, whereas more resource-intensive dietary structures may impose stronger constraints on land-use allocation and increase the risk of supply–demand imbalance [24]. Under such conditions, maintaining a land-use structure with sufficient cropland support becomes more important for sustaining regional stability and development [25].

At the regional level, Jiangsu is located in the lower Yangtze River and the core of the Yangtze River Delta, characterized by high development intensity, significant population and industrial agglomeration, and a typical plain river-network setting with dense water systems and numerous lakes [26]. As a result, its land-use structure is highly constrained and strongly coupled, especially through interactions among cultivated land, construction land, and water bodies. Studies on rapidly urbanizing plain river-network regions have shown that river systems tend to simplify during urban expansion, with tributaries declining significantly [27]. Because the connectivity and regulatory functions of water systems are highly sensitive to urbanization, treating water bodies as flexible land reserves may generate ecological service risks [28]. Therefore, in economically developed and water-network-dense regions such as Jiangsu, cropland pressure associated with dietary upgrading concerns not only agricultural space, but may also intensify conflicts between cropland compensation and urban expansion.

Based on the above considerations, this study develops an integrated analytical framework that incorporates both the demand side and the supply side into a unified system. On the demand side, the analysis centers on the evolution of dietary diversity among residents in Jiangsu Province from 1990 to 2023, constructs multiple dietary structure scenarios, and projects their trajectories to 2035 and 2050. These dietary scenarios are then converted into corresponding cropland demand for different food categories. On the supply side, the study considers land-system evolution under urban expansion and multiple development constraints, including natural development, cultivated land protection, ecological protection, and economic development scenarios. Through spatially explicit land-use simulation, it captures how rapid urban economic growth, particularly the expansion of construction land, reshapes land-use structure and compresses both cultivated land and ecological space. The cropland demand derived from different dietary scenarios is further treated as an external pressure on the land-structure system and incorporated into land-use simulations under four development scenarios. The resulting land-use structures are then compared with the baseline simulations under the same four scenarios in order to examine how supply–demand configurations under different land-use development objectives respond to changes in dietary structure. Although such pressures may not necessarily alter the overall direction of urban expansion, they may generate feedback through mechanisms such as cultivated land security and substitution among elastic land types, including grassland, water bodies, and unused land, as well as through the competitive reallocation between cultivated land and construction land.

Accordingly, this study proposes an analytical framework that links urban economic growth and consumption upgrading, dietary diversification, changes in cropland demand intensity, adjustment of land-use supply structure, responses of land patterns and conversion processes, and the resulting supply–demand matching and spatial trade-offs. Through multi-scenario coupled simulation, the study investigates the robustness of cropland supply–demand relationships and their spatial characteristics under different dietary pathways and development preferences. The aim is to provide both explanation and decision support for the coordinated optimization of cultivated land protection, ecological security, and dietary transition in rapidly developing regions. By adopting an integrated demand–supply–feedback systems perspective, this study seeks to explore the dual pressures of food security and supply–demand balance faced by economically advanced regions under dietary transition, and to provide an analytical basis for regional governance of supply–demand balance. The core concern of this study is not the broad reduction of rural production land per se, but rather the structural mismatch between cropland demand and cropland supply arising from the combined effects of dietary upgrading, urban expansion, and ecological constraints. The main objectives are: (i) to examine the dual pressures of food security and supply–demand balance faced by economically developed regions in the context of dietary transition; and (ii) to identify the basis for regional governance aimed at maintaining supply–demand balance. Based on this analytical framework, the study addresses the following research questions:

(1) Under dietary transition, what differences in cropland demand are generated by different dietary structure scenarios in Jiangsu Province, and what are their historical trajectories and future trends?
(2) Do different land-use development scenarios exhibit systematic differences in their capacity to accommodate diversified dietary demand, and which scenario is more conducive to maintaining the stability of cropland supply–demand matching?
(3) When dietary demand is incorporated into land-use simulation as an external constraint, through what types of land conversion, evolutionary pathways, and spatial hotspot responses will the land system feedback to different dietary scenarios?

Based on these questions, the study proposes the following hypotheses:

**H1.**  *Different dietary structure scenarios correspond to significant differences in both the scale and internal composition of cropland demand. As dietary patterns evolve from staple-dominated structures toward more diversified and resource-intensive forms, cropland demand pressure may gradually shift from being driven primarily by staple food demand to being increasingly influenced by animal-based foods and other high-value food products, thereby intensifying regional supply–demand pressure.*

**H2.**  *Different land-use development scenarios differ significantly in their capacity to accommodate dietary demand. Among them, the cultivated land protection scenario is expected to show greater stability in maintaining cropland supply–demand balance, whereas the natural development, ecological protection, and economic development scenarios are likely to have more limited capacity to support high-demand dietary patterns.*

**H3.**  *High-demand dietary scenarios are expected to intensify land-use adjustment within the land system and produce stronger spatial responses. Under such constraints, the land system may respond through more pronounced structural reallocation among cultivated land, construction land, water bodies, and other elastic land categories; in some scenarios, it may also exhibit reduced elasticity in construction land expansion.*

To address the above research questions and hypotheses, the analysis is conducted from the demand side, the supply side, and the feedback side. For H1, the cropland footprint approach and an LSTM forecasting model are used to estimate historical cropland demand under five dietary structure scenarios and to project future cropland demand for 2035 and 2050. For H2, the GeoSOS-FLUS model is employed to simulate cropland supply under four land-use development scenarios and to evaluate the supply–demand matching relationship between land supply and different dietary demands. For H3, diet-driven cropland demand is incorporated into the land-use simulation process as an external constraint in order to analyze land-category conversion, evolutionary pathways, and spatial hotspot responses under different scenario combinations.

## 2. Materials and Methods

### 2.1. Theoretical Framework

Before the separation of industry and agriculture became firmly established, urban and rural areas were more mutually embedded, and urban food supply relied heavily on nearby production, especially for perishable agricultural products with strong locational requirements, resulting in a localized production–distribution pattern that depended closely on surrounding cultivated land resources [29]. With rapid socioeconomic development, however, cultivated land around cities has been continuously lost, threatening the local supply of fresh agricultural products [4]. Rising opportunity costs of farming and growing income risk have further reduced the efficiency of cultivated land-use, in some cases even leading to land abandonment or fallowing [30]. At the same time, the production of meat, eggs, milk, vegetables, and other agricultural products has often failed to keep pace with rapid urban population growth and the continuous upgrading of food consumption patterns, resulting in increasingly tight effective supply [13]. In the short term, these tensions were alleviated by drawing more strongly on external markets and extra-local supply, but this also promoted the increasing basification, standardization, and spatial separation of agricultural production and distribution [31]. With deeper market integration following China’s accession to the World Trade Organization, local small-scale marketing channels faced stronger competition, while agricultural production became more integrated into wider supply chains and increasingly homogenized [31]. Under such conditions, a locally specialized and relatively single agricultural production structure has become increasingly unable to satisfy diversified urban food demand, and some large- and medium-sized cities have become more dependent on imports and extra-local supply for important agricultural products, showing a clear trend toward the delocalization of food systems [32]. Although this delocalized production–distribution pattern has maximized the capacity of cities to meet rising food demand, it has also raised concerns about the long-term sustainability of food systems as well as their short-term resilience and adaptive capacity [33]. Against this background, the idea of relocalization has gained importance. When extended from the broader re-embedding of human–land relations to cultivated land-use in urban regions, relocalization can be understood as a transition centered on urban–rural integration and symbiosis, in which scientific land-use planning helps foster short agricultural supply chains and strengthen local self-provisioning capacity [34]. The shift from delocalized food systems to a relocalization-oriented transition is shaped not only by changes in China’s food security strategy, urban–rural relations, and agricultural development philosophy, but also by the objective need to adapt to increasingly volatile global conditions [35]. The relocalization of cultivated land-use in urban regions aims to disperse production and transport risks, alleviate local supply–demand tensions, and improve the capacity to withstand short-term disruptions by building regional mechanisms for agricultural product security. At the same time, by diversifying modes of agricultural management, it may help to ease the long-term pressures faced by food systems and thus contribute to the construction of resilient food systems [36]. However, relocalization does not imply a simple return to traditional local self-sufficiency. Rather, it seeks to re-coordinate production–distribution relations and promote the sustainable development of food systems by leveraging the comparative resource advantages of specific regions [32].

Therefore, within regional food–nutrition systems, cropland can be regarded as a foundational and policy-regulatable asset that performs both bottom-line security and structural adjustment functions [37]. By contrast, market circulation and external imports more often act as short-term stabilizers and cross-regional balancing mechanisms [38]. When food and nutrition demand changes, the signal is first reflected in markets and then transmitted through prices, circulation, and trade to the cropland system, prompting adjustments in supply structure and generating a multilevel transmission pathway from shifts in demand structure to market rebalancing, interregional coordination, and cropland restructuring [9]. Cropland is not the sole source of nutritional supply, but it constitutes the bottom-line guarantee for staple-food and feed-grain security, whereas interregional circulation and trade mainly serve to complement and fine-tune the food and nutrition structure [39]. Diversified supply and cultivated land protection are mutually reinforcing. In China, cultivated land protection can be understood as gradually evolving from a type-oriented protection model focused on quantity, quality, and ecology toward a multifunctional and more function-oriented, multi-objective protection regime, while diversified demand in turn reshapes cropland patterns, production modes, and land functions, thereby driving cropland-use transition and institutional innovation [40]. Accordingly, this study focuses primarily on the matching relationship between cropland demand induced by dietary upgrading and the local supply capacity of the region.

**Figure 1:**
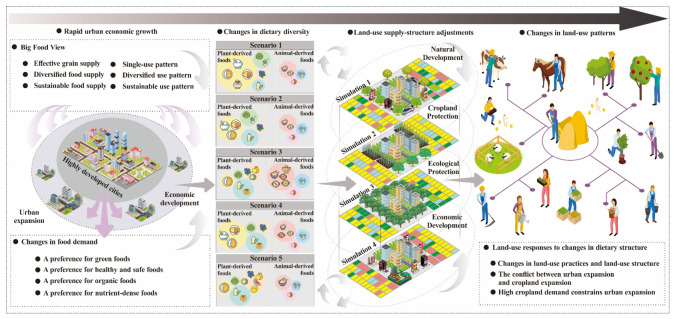
Theoretical framework.

Rising household income has driven a shift in dietary consumption from meeting basic food needs to pursuing better dietary quality [41]. Food security has shifted from a grain-centered focus to a diversified portfolio encompassing staple grains, animal-source foods, dairy, and fresh produce [8]. This shift has significantly changed residents’ dietary structures. On the demand side, the reduction in staple food consumption and the increase in meat consumption indicate that meat, dairy, and aquatic products can partially replace staple foods [42]. However, this also stimulates the demand for feed grains, transforming the contradiction of food security from one of quantity to a dual contradiction of both quantity and structure [43]. Moreover, dietary diversity has led not only to a surge in demand for animal-based foods but also to an increase in demand for vegetables, fruits, and other non-grain foods. As a result, the focus of food security has shifted from ensuring the supply of staple foods to ensuring the overall supply of staple foods, feed grains, and other important agricultural products [44]. On the supply side, the expansion of construction land, ecological protection constraints, and the cultivated land protection red line will further alter the land supply structure, subjecting cultivated land supply to multiple quantity, spatial, and ecological constraints [45]. Existing research suggests that income growth and urbanization drive the transformation of dietary patterns from plant-based staples to high-fat, high-animal food diets with a more diverse structure, which significantly increases land occupation and environmental pressure [46]. In contrast, transitioning to healthier, more plant-based diets can effectively reduce the burden on land and the environment. At the same time, the impact of dietary structure on land demand is not solely reflected in increased consumption; more crucially, it lies in the structural changes, primarily through mechanisms such as feed conversion, yield per unit area, and crop rotation, which alter cultivated land-use per capita, thereby reshaping regional cultivated land demand intensity and pressure transmission pathways [47]. From a long-term development perspective, by 2035, China’s ecological civilization construction goals will aim to comprehensively improve material, political, spiritual, social, and ecological civilization [48]. A green development approach and lifestyle will be fully established, with harmonious coexistence between humans and nature, and the modernization of the national governance system and capabilities in the ecological environment will be fully realized, contributing to the creation of a beautiful China. Compared to the 2035 vision, the 2050 vision emphasizes the attributes of a socialist modernized strong nation. By 2050, China is expected to have comprehensively built an ecologically prosperous civilized society, leading the construction of a human community in the era of ecological civilization [49]. This will include zero emissions of pollution, removal of existing pollution stocks, and restoration of natural states, with ecosystems becoming more resilient, ecological dividends continuously released, and a balanced and harmonious relationship between humans and nature, achieving ecological prosperity and fully realizing the modernization of the national governance system and capabilities in ecological environmental areas [50]. Therefore, at the critical time points of 2035 and 2050, addressing the balance between dietary structure upgrading and cultivated land-use structure supply and demand is urgent, given the aspirations for a high-quality life for the people and the major national development plans (Figure 1).

### 2.2. Study Area

**Figure 2:**
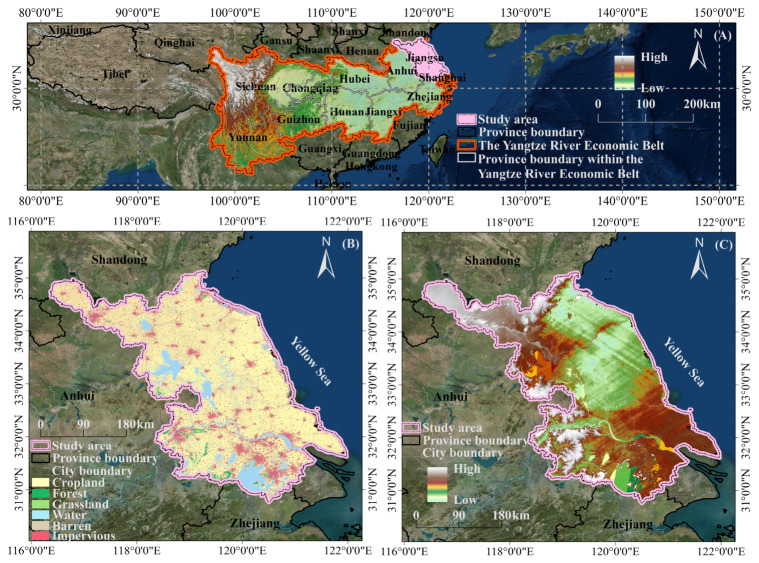
Map of the study area. (**A**) Location of the study area in the Yangtze River Economic Belt; (**B**) Land-use of the study area; (**C**) Elevation of the study area.

**Figure 3:**
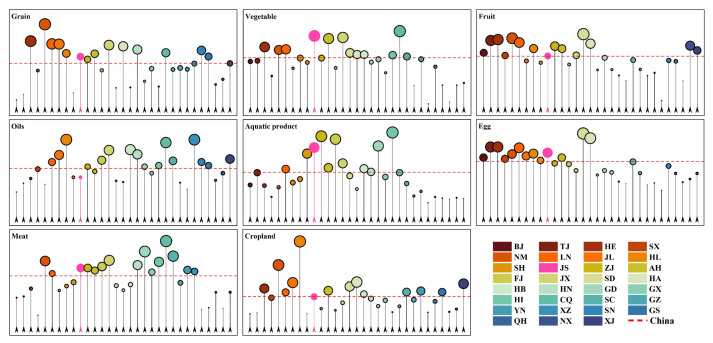
Per Capita Consumption of Different Food Categories and Total Cultivated Land Area by Province in China.

This study selects Jiangsu Province as a typical case area, located at a key geographical position within the Yangtze River Economic Belt (downstream) and the core region of the Yangtze River Delta. It is bordered by the Yellow Sea to the east, Shanghai and Zhejiang to the south, Anhui to the west, and Shandong to the north. Jiangsu serves as an important hub connecting the coastal developed provinces of China and the eastern part of the Yangtze River Economic Belt. The study area is primarily composed of low, flat plains with an intensive water network. The river and lake systems are well-developed, with minor topographic variations, although there are also local low mountains and hills. From the perspective of land-use patterns, cultivated land still dominates the landscape, extensively distributed across the northern and central plains of Jiangsu. Construction land is highly concentrated in the urban belt along the Yangtze River and in the southern parts of the province, showing a trend of continuous expansion. Water areas also occupy a significant proportion and are continuously distributed, forming a distinctive river network landscape (Figure 2). According to the 2025 China Statistical Yearbook (Figure 3), Jiangsu in 2024 can be characterized by relatively high food consumption and only moderate cultivated land availability. Specifically, when examining per capita consumption of different foods, Jiangsu residents consume significantly more vegetables (131.6 kg), aquatic products (26.8 kg), eggs (17.4 kg), and meat (41.8 kg) compared to the national average (108.6, 15.1, 14.1, and 38.1 kg, respectively). Among these, vegetable and aquatic product consumption is 21% and 77% higher than the national averages, while egg and meat consumption is approximately 23% and 10% higher, respectively. Grain consumption (131.3 kg) is slightly higher than the national average (124.4 kg), and fruit consumption (62.0 kg) is almost the same as the national average (61.6 kg). However, plant oil consumption (8.2 kg) is lower than the national level (9.7 kg). In terms of national rankings, Jiangsu ranks 2nd in vegetable consumption, 5th in aquatic product consumption, and 6th in egg consumption, reflecting the typical dietary structure of a developed coastal region characterized by relatively high consumption of vegetables, aquatic products, and eggs. Compared to other economically developed areas within the Yangtze River Economic Belt, Jiangsu has the highest per capita egg consumption (17.4 kg) in the region. It ranks 2nd in both per capita consumption of aquatic products and vegetables, with aquatic products second only to Zhejiang (32.1 kg) and vegetables second only to Chongqing (136.8 kg). This indicates that Jiangsu is representative of the relatively diverse and high-level food consumption patterns found in economically developed regions. When compared to other developed regions like Shanghai and Zhejiang, Jiangsu’s vegetable consumption is significantly higher than both Shanghai (102.8 kg) and Zhejiang (107.6 kg). Its aquatic product consumption is higher than Shanghai (23.9 kg) but lower than Zhejiang (32.1 kg), and its egg consumption is noticeably higher than both Shanghai (14.5 kg) and Zhejiang (13.4 kg). This reflects Jiangsu’s distinctive combination of plain river-network geography, coastal resource endowments, and relatively high consumption levels within the Yangtze River Delta. At the same time, Jiangsu’s total cultivated land area is about 4.14 million hectares, placing it in the middle range nationally (ranked 15th). Within the Yangtze River Economic Belt, this is lower than provinces like Anhui, Yunnan, Sichuan, and Hubei, making it a typical case in the eastern Yangtze River Economic Belt, where strong consumption demand coexists with relatively limited cultivated land supply. Thus, Jiangsu is a representative case for understanding the tension between upgrading food demand and supply-side land constraints faced by the developed eastern regions of the Yangtze River Economic Belt during dietary transition.

### 2.3. Data Source

The data used in this study mainly include physical geographic data, locational vector data, remote sensing imagery, and socioeconomic statistical data. Land-use data were obtained from the 30 m resolution China Land Use/Cover Remote Sensing Monitoring Dataset released by the Resource and Environment Science and Data Center (RESDC) of the Chinese Academy of Sciences. According to the needs of this study, land-use data for 1990, 2000, 2010, 2020, and 2023 were selected for land-use change analysis and simulation. Socioeconomic statistical data were primarily collected from the China Statistical Yearbook (1991–2024 editions) and the Jiangsu Statistical Yearbook (1991–2024 editions). Regarding dietary data, and based on the food consumption characteristics of residents in Jiangsu Province, food items were classified into seven categories: grains, vegetables, fruits and melons, vegetable oil, livestock and poultry meat (including pork, beef and mutton, and poultry), eggs, and aquatic products. Data for the current dietary structure scenario were mainly derived from the statistics on residents’ food consumption reported in the China Statistical Yearbook and the Jiangsu Statistical Yearbook from 1990 to 2023. The balanced dietary structure scenario was constructed based on the recommended intake levels for different food categories provided in the Dietary Guidelines for Chinese Residents (2022). Data for the dietary structure scenarios of the United States, Japan, and Greece were mainly obtained from FAOSTAT, using the average food balance data for 2021–2023.

### 2.4. Estimation and Simulation of Cultivated Land Demand Under Different Dietary Structure Scenarios

#### 2.4.1. Estimating Cultivated Land Demand Under Different Dietary Structure Scenarios

The ecological footprint approach is widely regarded as an important tool for assessing resource use and sustainability. The ecological footprint concept, proposed by Rees, was developed to quantify the natural resource consumption associated with a given population under a particular consumption pattern, as well as the resulting environmental impacts [51]. Through subsequent development, a relatively mature and comprehensive ecological footprint accounting framework has been established. With a primary focus on the demand side, the cultivated land ecological footprint of food consumption is a specific application of the ecological footprint concept. It refers to the area of cultivated land required, over a given period, to meet the food consumption needs of a specific population (including grains, vegetables, fruits, oils, aquatic products, eggs, meat). By converting residents’ food consumption into the corresponding cultivated land demand area, this metric captures the pressure exerted by the population on land resources. It is intended to provide an intuitive reflection of how food consumption affects land resources and the environment, as well as the level of sustainable development. Accordingly, drawing on previous studies, the cultivated land footprint method is used to estimate the cultivated land area required for different food categories (LRF), using the following formula [14]:

(1) $$L_{P}=\sum_{i=1}^{n}\frac{x_{i}}{E_{i}\times F_{i}}\times \frac{1}{c}$$

(2) $$L_{A}=\sum_{i=1}^{n}\frac{x_{i}}{E_{i}\times F_{i}}\times \frac{H_{i}}{c}$$

where $L_{P}$ denotes the land demand for plant-based foods, $L_{A}$ denotes the land demand for animal-based foods; $x_{i}$ is the quantity of food consumed; $E_{i}$ is the conversion rate of the food during processing (Table 1); $F_{i}$ is the yield per unit area of the corresponding raw crop; $H_{i}$ is the amount of feed grain required to produce one unit of animal-based food (aquatic products: 1.06 kg/kg, eggs: 2.19 kg/kg, meat: 3.09 kg/kg); c is the multiple cropping index.

Based on the FAOSTAT database and the actual per capita food consumption of residents in Jiangsu Province, this study defines five dietary structure scenarios (Table 2). These scenarios do not assume that the region will directly replicate the actual dietary pattern of any specific country in the future. Rather, they use reference dietary prototypes with clearly differentiated food composition and resource demand to construct a comparative framework spanning the observed dietary trajectory, a nutritionally balanced pathway, and more resource-intensive or alternative dietary pathways [13,55,56]. The scenario selection was guided by three main considerations. First, the data source is unified and comparable, as the international reference scenarios were derived from FAOSTAT Food Balances data [56]. Second, the selected scenarios display clear gradient differences in the consumption of plant-based foods, animal-source foods, oils, fruits, and vegetables, which makes it possible to more effectively identify the sensitivity of cropland demand to dietary change [13,55]. Third, these scenarios provide meaningful reference pathways for the potential upgrading of dietary patterns among Chinese residents [57]. Among them, the current dietary structure scenario (Scenario 1) is constructed on the basis of the actual per capita food consumption of Jiangsu residents and is used to represent the observed trajectory of dietary change. The balanced dietary scenario (Scenario 2) is established with reference to the Dietary Guidelines for Chinese Residents (2022) and serves as a benchmark scenario oriented toward nutritional balance [57]. The U.S. dietary structure scenario (Scenario 3) is used to represent a dietary prototype characterized by high consumption of animal-source foods, high oil intake, and relatively high resource demand, and is therefore treated as the upper-pressure boundary for cropland demand [55]. The Japanese dietary structure scenario (Scenario 4) is selected because Japan shares a broader East Asian dietary cultural background with China and exhibits a comparatively balanced food composition; it is therefore used as a reference scenario for dietary transition in developed East Asian regions [58]. The Greek dietary structure scenario (Scenario 5) is used to represent a typical Mediterranean dietary prototype, characterized by relatively high shares of vegetables, fruits, and plant oils and relatively low red-meat intake, and is employed to examine the implications of a fruit- and vegetable-intensive dietary pathway for cropland demand [59]. Therefore, the five dietary scenarios in this study should be understood as stylized representations of multiple possible directions for the future evolution of dietary structure in Jiangsu, rather than as a simple transplantation of national dietary patterns from other countries. Their function is to provide a set of clearly differentiated and comparable scenario boundaries for cropland demand estimation, supply–demand matching identification, and land-system feedback analysis in Jiangsu Province.

#### 2.4.2. Predictive Simulation of Cultivated Land Demand Under Different Dietary Structure Scenarios

**Figure 4:**
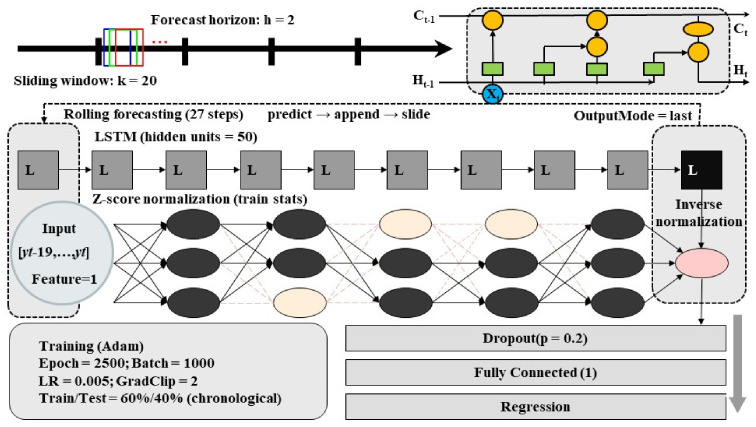
Conceptual Diagram of the LSTM Forecasting Simulation.

This study employs a Long Short-Term Memory (LSTM) network to construct a univariate time-series forecasting model. Model development and training were implemented in MATLAB (R2024a, MathWorks, Natick, MA, USA). LSTM was selected because the annual cropland-demand-related series used in this study exhibit temporal dependence, nonlinear variation, and potential lagged effects that are difficult to represent adequately through simple linear extrapolation. Compared with more conventional forecasting approaches, LSTM provides greater flexibility in capturing nonlinear temporal relationships without imposing a strict functional-form assumption. At the same time, given the limited length of the annual time series, the forecasts in this study are interpreted primarily as internally comparable scenario projections rather than precise long-term predictions. As an improved form of the Recurrent Neural Network (RNN), LSTM uses an input gate, forget gate, output gate, and a cell state to selectively retain and update information over time. In this way, it effectively mitigates the vanishing and exploding gradient problems typical of conventional RNNs in long-sequence modeling, and enhances the representation of long-range dependencies [60,61]. First, the target series *yt* is converted into supervised-learning samples using a sliding window: with window length *k* = 20 and forecast horizon *h* = 2 (i.e., using [*yt* − 19, …, *yt*] to predict *y_t_* + 2). Samples are split into a training set and a test set in chronological order (60% for training) to avoid information leakage. To improve convergence and ensure comparability across variables, Z-score standardization is applied separately to inputs and outputs. The standardization parameters (mean and standard deviation) are estimated using only the training set, and then applied to the training set, test set, and future forecasts; model outputs are subsequently inverse-transformed to recover the original scale. The network architecture is sequenceInputLayer (1)–lstmLayer (50, OutputMode = last)–dropout (0.2)–fullyConnected (1)–regressionLayer, where the final hidden state represents historical information and outputs an h-step-ahead point forecast (*h* = 2). Training uses the Adam optimizer with a maximum of 2500 iterations, an initial learning rate of 0.005, and MiniBatchSize = 1000. Given the limited number of supervised samples derived from the annual time series, this setting effectively corresponds to full-batch (or near full-batch) updates in practice. Gradient clipping is set to 2 to prevent exploding gradients. At the supervised-sample level, samples are randomly shuffled to improve training stability, while preserving the chronological train–test split. Model performance is evaluated on both the training and test sets using R^2^, MAE, MBE, nMAE%, nMBE%. Future projections are generated via an iterative rolling procedure for the next 27 years: at each step, a single forecast with *h* = 2 is produced from the current window, the predicted value is appended to the end of the window, and the window is updated by sliding forward; this process is repeated until the full forecast series is obtained. To reduce uncertainty arising from random initialization and batch sampling, the model is trained 500 times, and mean values are reported for both evaluation metrics and future forecasts to improve robustness (Figure 4).

According to Table 3, the model shows strong performance for population (test-set R^2^ = 0.991; nMAE = 0.23%; nMBE = 0.07%), indicating that it captures the long-term trend well. For cultivated land demand area by food category, test-set R^2^ remains relatively high (generally 0.870–0.986), while the relative errors are non-negligible (with nMAE varying by category), suggesting that the model is better suited to capturing temporal variation and scenario-wise contrasts than producing precise point estimates for every category. In particular, per capita consumption series exhibit substantial heterogeneity (e.g., fruits, plant oils, and eggs), consistent with the influence of exogenous drivers (dietary transitions, prices/income dynamics, and potential structural breaks) that are not represented in a univariate framework. Therefore, the forecasts are used primarily as consistent, internally comparable inputs for multi-scenario coupling and sensitivity analysis, and results should be interpreted with appropriate caution regarding absolute magnitudes. Although prediction uncertainty remains for several original consumption series, the transformed food-category-specific cropland demand area shows a more stable comparative structure, which is the main reason why the LSTM outputs are retained as comparative inputs in the coupled framework.

### 2.5. Simulation of Land-Use Structure Under Different Development Scenarios

GeoSOS-FLUS software (Future Land Use Simulation model, available at geosimulation.cn/FLUS.html, accessed on 2 January 2026) was used for multi-class land-use change scenario simulation. GeoSOS-FLUS is a simulation platform developed to model future land-use change by coupling human and natural effects. It uses LUCC data together with driving factors to simulate future land-use changes under different influencing conditions [62].

(1) Simulation of the probability estimation module. Based on an artificial neural network (ANN) algorithm, this module includes both training and prediction stages and consists of an input layer, a hidden layer, and an output layer. The calculation is as follows:

(3) $$P(p,k,t)=\sum_{j}w_{jk}\times \frac{1}{1+e^{-net_{j}(p,t)}}$$

where P(p,k,t) denotes the suitability probability of land-use type *k* for grid cell *p* at time *t*; $w_{jk}$ is the weight between hidden neuron *j* and output node *k*; net is the net input of a hidden-layer neuron; e is the natural constant; and $-net_{j}(p,t)$ represents the signal received by neuron *j* at grid cell *p* at time *t*.

(2) Cellular automata based on an adaptive inertia mechanism. Using multi-class LUCC raster data, land-use scenarios are simulated by integrating suitability probabilities, the expected change (quantity) for each LUCC type, a conversion cost matrix, and neighborhood factor parameters. The adaptive inertia coefficient is calculated as follows:

(4) $$I_{k}^{t}\left\{\begin{array}{c} I_{k}^{t-1},\;\;\;\;\;\;\;\;\;\;\;if\left|D_{k}^{t-1}\right|\leq \left|D_{k}^{t-2}\right|\\ I_{k}^{t-1}\times \frac{D_{k}^{t-2}}{D_{k}^{t-1}},\;ifD_{k}^{t-1}<D_{k}^{t-2}<0\\ I_{k}^{t-1}\times \frac{D_{k}^{t-1}}{D_{k}^{t-2}},\;if0<D_{k}^{t-2}<D_{k}^{t-1} \end{array}\right.$$

where $I_{k}^{t}$ and $I_{k}^{t-1}$ are the inertia coefficients of land-use type *k* at iteration *t* and *t* − 1, respectively; $D_{k}^{t-1}$ and $D_{k}^{t-2}$ denote the area differences between land demand and the actual area of land-use type *k* at iterations *t* – 1 and *t* − 2, respectively.

(3) Multi-scenario settings. Multi-scenario land-use simulation requires estimating, for each specific grid cell, the integrated probability of transitioning to different land-use types. The conversion cost reflects the difficulty of converting between land-use types and is assigned a value of 0 or 1. Because future urban land-use change is subject to different constraints across scenarios, different restriction conditions must be introduced when modeling land-use change in order to control urban spatial form under each scenario in the target years. In this study, relevant planning constraints are incorporated into simulations of the spatial evolution of Jiangsu’s land-use structure. Based on four scenarios—natural development, cultivated land protection, ecological protection, and economic development—four land-use development modes are specified (Simulation 1–4). Setting the conversion cost matrix facilitates projecting land-type transitions under different contexts, thereby generating the expansion patterns of various land-use types under different development modes (Table 4).

The neighborhood weight of a land-use type represents the strength of its spatial expansion capability. The parameter ranges from 0 to 1, and values closer to 1 indicate stronger spatial expansibility (Table 5). Drawing on relevant studies and the specific conditions of Jiangsu Province, this study pre-sets neighborhood weights for each land-use type and conducts simulation experiments. After repeated model calibration, the final neighborhood weights for each land-use type under different development simulations in Jiangsu are determined. A random sampling approach is adopted: 10% of valid raster cells are selected based on the total sample size. The resulting Kappa coefficients for all simulations exceed 0.75, indicating high simulation accuracy. Therefore, the parameter settings are considered reasonable (Table 6).

(4) Driving factors [63]. Based on the conditions in Jiangsu Province and considering natural, climatic, socio-economic, and locational determinants, fourteen driving factors are selected: elevation (DEM), NDVI, topsoil total nitrogen, topsoil organic carbon content, topsoil pH, topsoil clay content, topsoil silt content, mean annual precipitation, GDP, population density, distance to the river network, distance to railways, distance to expressways, and distance to urban squares

## 3. Results

### 3.1. Demand Side—Changes in Dietary Structure Diversity

#### 3.1.1. Food Consumption Changes in Different Dietary Structure Scenarios over 1990–2023

From 1990 to 2023, the food consumption structure of residents in Jiangsu Province showed a clear transition from a staple grain-oriented pattern to a more diversified dietary structure. This transition was marked by a continuous decline in grain consumption and a general increase in the consumption of fruits and melons, vegetable oil, aquatic products, eggs, and livestock and poultry meat, while vegetable consumption remained relatively stable. These trends indicate that dietary demand in Jiangsu has gradually shifted from an emphasis on staple food sufficiency toward a more diversified and nutrition-oriented pattern. The Pettitt change-point test, combined with the Mann–Kendall trend test and Sen’s slope estimator, shows that structural turning points were mainly concentrated in 2004–2007 and 2011–2013, suggesting that the period from the early-to-mid 2000s to the early 2010s was the most significant stage of dietary restructuring (Table 7). Grain consumption declined significantly throughout the study period, with the most rapid decrease occurring between 2008 and 2013, followed by a phase of low-level fluctuation after 2014. By contrast, fruits and melons, eggs, aquatic products, and livestock and poultry meat all exhibited significant upward trends, although growth rates differed across stages. Vegetable oil consumption increased overall and was characterized more by stepwise structural growth, whereas vegetable consumption showed only gradual adjustment without a stable unidirectional trend (Table 8).

**Figure 5:**
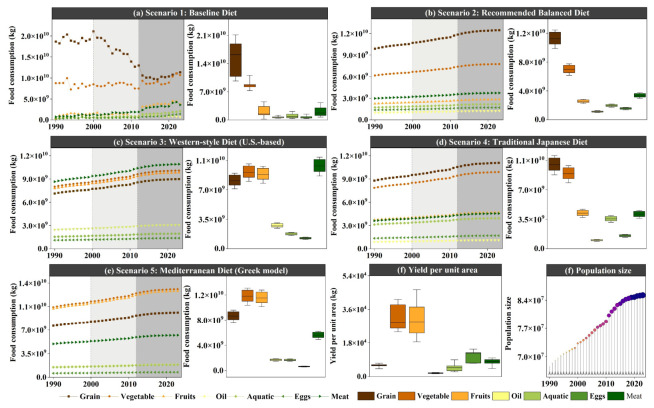
Food Consumption under Different Scenarios.

Based on these statistical results and the overall patterns in the consumption series, the evolution of Jiangsu residents’ food consumption can be divided into three stages: 1990–2000, a period of stable and relatively high grain consumption; 2000–2012, a period of pronounced dietary transition; and 2012–2023, a period of diversification and relatively stable growth (Figure 5). Among these, the second stage was the most important phase of structural adjustment, during which grain consumption declined markedly while non-grain food consumption increased continuously, driving the dietary structure from a staple-oriented pattern toward a more diversified configuration. A comparison with the parameter settings of the different dietary scenarios further suggests that Jiangsu’s current consumption structure is relatively close to the balanced dietary scenario and the Japanese dietary scenario. However, compared with the U.S. and Greek dietary scenarios, grain consumption in Jiangsu still accounts for a relatively large share, while the shares of fruit, vegetable, and meat consumption remain adjustable. Overall, although the dietary structure of Jiangsu residents has undergone substantial transformation, it remains in a transitional stage from a traditional staple-based diet toward a higher-quality and more diversified dietary pattern.

#### 3.1.2. Changes in Land Area Demand Under Different Dietary Structure Scenarios over the Past 30 Years

From 1990 to 2023, the land area required to satisfy food demand under the five dietary scenarios exhibited broadly similar stage-specific dynamics. Specifically, 1990–2000 was a period of gradual decline, 2000–2012 was a period of rapid adjustment, and 2012–2023 entered a relatively stable phase (Table 9 and Table 10). Despite these shared temporal patterns, the overall scale of land demand differed significantly across scenarios. Pairwise comparisons showed that all five scenarios differed from one another at statistically significant levels, with a stable overall ranking of Scenario 3 > Scenario 5 > Scenario 4 > Scenario 2 > Scenario 1 (Table 11). This indicates that although different dietary structures were embedded in a similar temporal context, differences in food composition produced persistently divergent levels of land demand, and resource-intensive dietary structures consistently imposed greater pressure on cropland demand.

A closer examination of the internal structure of each scenario reveals marked differences in the food categories that dominated demand-related land-use. In Scenario 1, land demand was still primarily driven by grain consumption, followed by vegetables, vegetable oil, fruits and melons, meat, aquatic products, and eggs. In Scenario 2, grain remained the largest component, but vegetable oil and meat moved up substantially, yielding the order of grain, vegetable oil, meat, vegetables, fruits and melons, aquatic products, and eggs. Scenario 4 showed a similar pattern to Scenario 2, with a relatively balanced structure in which grain, vegetable oil, and meat occupied the leading positions. By contrast, Scenario 3 displayed a clear structural shift, with land demand dominated by vegetable oil, meat, and fruits and melons, while grain moved to a secondary position. Scenario 5 was characterized by the dominance of fruits and melons, vegetable oil, and meat, with grain ranking only in the middle-to-lower range. These results suggest that Scenarios 1, 2, and 4 still retained strong features of a grain-dominated or grain–vegetable oil–meat co-dominated structure, whereas Scenarios 3 and 5 exhibited a distinctly non-grain-dominated pattern.

From the perspective of cross-scenario differences by individual food category, variations in total land demand were not driven by simultaneous expansion across all food types, but were mainly shaped by four categories: grain, vegetable oil, fruits and melons, and livestock and poultry meat (Table 12 and Table 13). Grain-related land demand remained central in Scenarios 1, 2, and 4, constituting the basic source of land demand under more traditional dietary structures. However, in Scenarios 3 and 5, the relative importance of grain declined substantially, indicating a weaker dependence on staple foods under diets characterized by higher consumption of animal-source foods or fruits and vegetables. In contrast, vegetable oil and livestock and poultry meat moved forward significantly in Scenarios 2–5, especially in Scenario 3, where they became the dominant components. This suggests that high oil intake and high consumption of animal-source foods are key factors underlying the widening gap in total land demand across scenarios. Land demand associated with fruits and melons was most prominent in Scenario 5 and also accounted for a relatively large share in Scenario 3, indicating that fruit- and vegetable-intensive dietary structures can further intensify non-grain land-use pressure. By comparison, aquatic products and eggs consistently ranked lower across all five scenarios and contributed relatively little to explaining differences in total demand area, functioning more as supplementary components of dietary structure than as determinative components.

**Figure 6:**
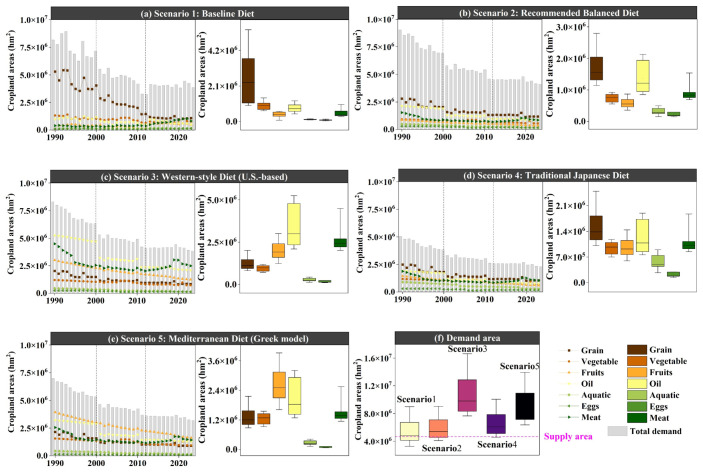
Food Demand Area under Different Scenarios.

In terms of stage-specific change, although all scenarios experienced similar temporal turning points, their later structural trajectories diverged. In Scenario 1 and 2, total demand area declined during the first two stages and the internal structure changed only moderately; however, in the third stage, land demand associated with meat increased markedly, and demand for animal-source foods gradually surpassed that for plant-based foods. This suggests that both the current dietary scenario and the balanced dietary scenario exhibit a tendency to shift from a staple-dominated structure toward one in which grain and meat are jointly important. By contrast, Scenario 3 and 5 maintained a structure dominated by vegetable oil, meat, and fruits and melons throughout the entire period. Although total demand area declined over time, their internal composition did not undergo any fundamental reversal. Scenario 4 occupied an intermediate position: on the one hand, it continued to rely on grain as a major support; on the other hand, vegetable oil, meat, and fruits and melons accounted for relatively balanced shares, reflecting both stability and transitional characteristics. Overall, the differences among the five dietary scenarios are reflected not only in which scenario generates a larger land demand area but also, more importantly, in which food categories dominate that demand. This suggests that the core issue in food-demand-related land-use change is not simply a quantitative increase or decrease in total area, but rather a process of land-demand reallocation induced by dietary restructuring (Figure 6).

#### 3.1.3. Forecast of Food Demand Area Under Different Dietary Structure Scenarios

**Figure 7:**
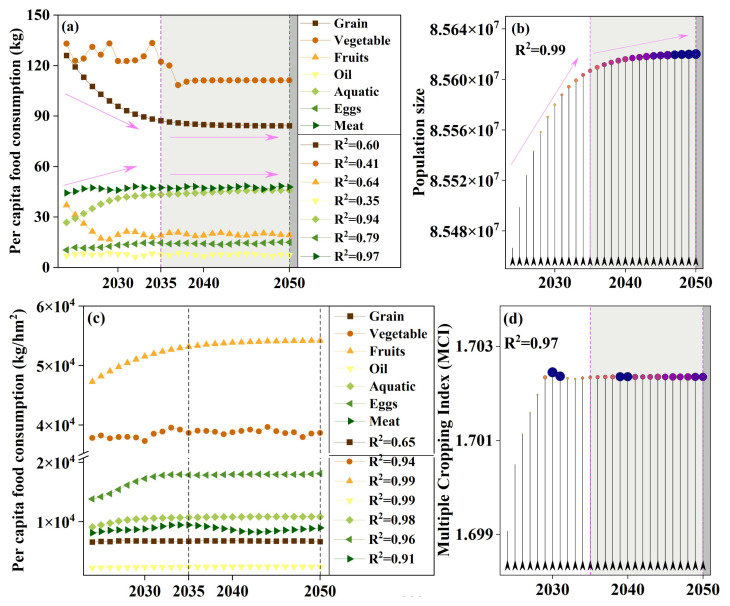
Predicted Results for Food Consumption, Population, Yield per Unit Area, and Multiple Cropping Index. (**a**) Forecast results for food consumption; (**b**) Forecast results for population size; (**c**) Forecast results for yield per unit area; (**d**) Forecast results for the multiple cropping index.

**Figure 8:**
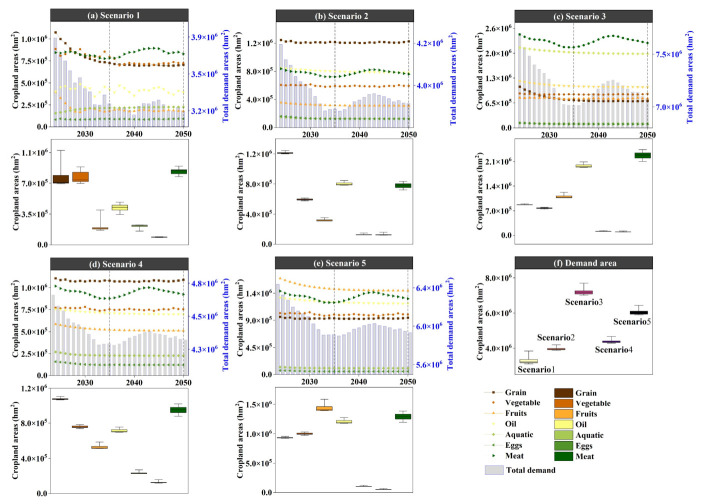
Predicted Results for Food Demand Area under Different Scenarios.

From the predicted food consumption, the overall order of food consumption from highest to lowest is as follows: vegetables, grains, meats, aquatic products, fruits, eggs, and plant oils. In terms of time variation, the food types showing a downward trend are grains, vegetables, and fruits, while the food types showing an upward trend are meats, aquatic products, eggs, and plant oils. All food types are expected to stabilize by 2035. The predicted population shows an increasing trend year by year, but it is expected to stabilize by 2035. The changes in per unit yield for different foods are not significant, with the order from highest to lowest being fruits, grains, eggs, aquatic products, meats, oils (Figure 7). The variation in the multiple cropping index is also relatively smooth. Based on the predicted food consumption, population, yield per unit, and multiple cropping index, the changes in demand area under different dietary structure scenarios are shown in the figure. In Scenario 1 (current food structure scenario), the top three food types in terms of demand area are meats, vegetables, and grains. Before 2035, the demand area for grains and vegetables is higher than that for meats, but after 2035, the demand area for meats gradually surpasses that for grains and vegetables, becoming the food type with the highest demand area. By 2050, the food demand areas, from highest to lowest, are meats, vegetables, grains, plant oils, aquatic products, fruits, and eggs. In Scenario 2 (balanced dietary structure scenario), the top three food types in terms of demand area are grains, plant oils, and meats. The changes in the demand area structure for this scenario are not significant in the future and show a relatively stable development. In Scenario 3 (U.S. dietary structure scenario), compared to Scenario 1 and 2, the demand area for meats significantly increases. The top three food types in terms of demand area are meats, plant oils, and fruits, with grains exiting the top three. Over time, the demand area for grains drops to second to last by 2035, then stabilizes. By 2050, the food demand areas, from highest to lowest, are meats, aquatic products, plant oils, vegetables, fruits, grains, and eggs. In Scenario 4 (Japanese dietary structure scenario), the top three food types in terms of demand area are grains, meats, and vegetables. The changes in the demand area structure for this scenario are not significant in the future, and it shows relatively stable development. In Scenario 5 (Greek dietary structure scenario), the top three food types in terms of demand area are fruits, meats, and plant oils. This scenario’s food demand is mainly driven by fruits, meats, and plant oils, with vegetables and grains following. The demand area for aquatic products and eggs is much lower. Overall (Figure 8), Scenario 3 has the highest demand area, followed by Scenario 5, Scenario 4, Scenario 2, and Scenario 1.

### 3.2. Supply Side—Land-Use Changes Under Multiple Scenarios

**Figure 9:**
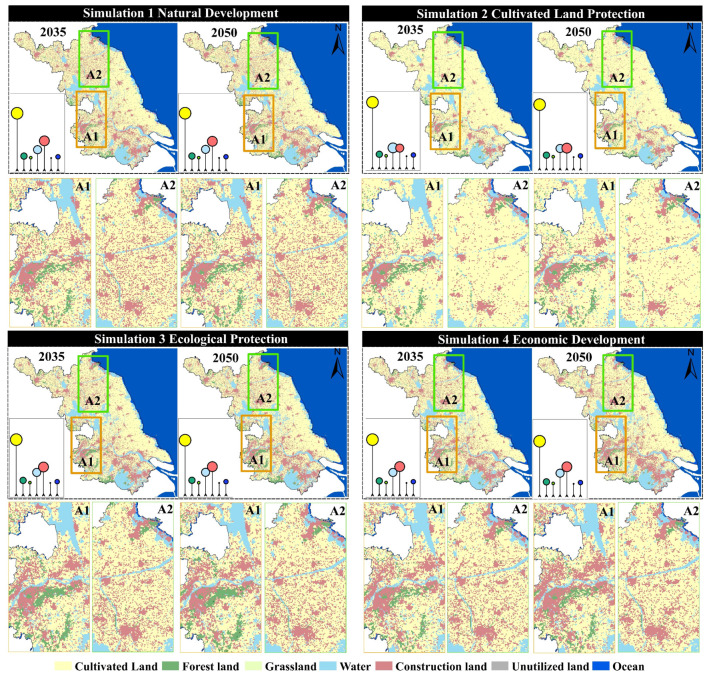
Land-Use Structure Simulation under Different Development Scenarios.

As shown in Figure 9, land-use in the four scenarios from 2035 to 2050 generally follows a common evolutionary direction of “expansion of construction land-pressure on cultivated land.” However, different development preferences significantly alter the intensity and spatial patterns of expansion. In the natural development scenario (Simulation 1), construction land continues to expand outward around existing urban patches, and it spreads in a linear pattern along major transportation corridors and river networks, leading to more pronounced fragmentation of cultivated land in urban transition zones. In areas like A1 and A2, there is a noticeable trend of construction land encroaching on surrounding cultivated land. In the cultivated land protection scenario (Simulation 2), the connectivity and integrity of cultivated land patches are significantly better than in the natural development scenario, and the expansion of construction land is more tightly constrained. New construction land tends to grow “compactly” at the edges of existing built-up areas, which slows the rate of conversion of cultivated land to construction land. In A1, the cultivated land patches become more concentrated, with less fragmentation caused by construction land. In the ecological protection scenario (Simulation 3), there is an expansion and aggregation of ecological land (forest/grassland), especially in ecologically sensitive areas or areas requiring ecological corridors. Forest and grassland patches become more connected with more stable boundaries. As a result, construction land expansion is most restrained, and some peripheral cultivated land may be converted to ecological land, enhancing the ecological foundation of the regional landscape. In contrast, the economic development scenario (Simulation 4) shows the most significant increase in construction land. Urban patches expand further, with a higher degree of contiguity, and more pressure to expand in core development areas such as A1. Cultivated land is most occupied, and landscape fragmentation is most pronounced. Meanwhile, water and ocean remain generally stable, with only slight type adjustments along certain shorelines and urban edges. Overall, the comparison of scenarios indicates that cultivated land protection effectively suppresses the conversion of “cultivated land–construction land” and enhances the connectivity of cultivated land. Ecological protection is more beneficial for enhancing the ecological foundation of forests and grasslands and stabilizing the landscape, while economic development leads to a higher intensity of construction land expansion, improving spatial capacity but bringing more significant risks of cultivated land occupation and landscape fragmentation.

### 3.3. Multi-Scenario Dietary Structure Cultivated Land Supply–Demand Matching

**Figure 10:**
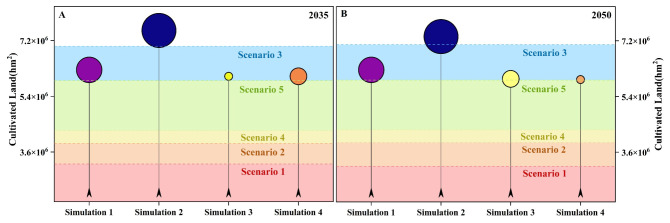
Land-Use Structure under Different Development Scenarios and Supply–Demand Matching with Different Dietary Structure Scenarios. (**A**) Supply–demand matching in 2035; (**B**) Supply–demand matching in 2050.

As shown in Figure 10, the cultivated land supply scale in 2035 and 2050 under the four land-use simulation scenarios (Simulation 1–4) exhibits a clear gradient matching relationship with the cultivated land demand ranges corresponding to the five dietary structure scenarios (Scenario 1–5). Overall, the cultivated land demand ranges from low to high as follows: Scenario 1 (current food structure), Scenario 2 (balanced dietary structure), Scenario 4 (Japanese dietary structure), Scenario 5 (Greek dietary structure), and Scenario 3 (U.S. dietary structure). Among these, the U.S. dietary scenario corresponds to the highest cultivated land demand and imposes the strongest supply constraint. Comparing different land-use scenarios, it is evident that the cultivated land protection scenario (Simulation 2) provides the highest cultivated land supply in both target years (2035 and 2050). The supply level in this scenario is sufficient to support the high demand of the U.S. dietary scenario (Scenario 3). In contrast, the natural development (Simulation 1), ecological protection (Simulation 3), and economic development (Simulation 4) scenarios generally provide cultivated land supply within the demand range of the Greek dietary scenario (Scenario 5), indicating that without an emphasis on cultivated land protection, the regional cultivated land supply has limited capacity to support high-resource-consuming diets (especially Scenario 3). In terms of temporal evolution, the ranking of cultivated land supply across different land-use scenarios from 2035 to 2050 remains generally stable (Simulation 2 provides the highest supply, with the other three scenarios being relatively close). However, under the economic development scenario (Simulation 4), the supply in 2050 slightly decreases, and the gap with high-demand dietary scenarios further expands. Overall, the results reveal that the emphasis on land-use development (whether or not “cultivated land protection” is prioritized) determines whether high cultivated land demand dietary scenarios can be supported. Additionally, dietary structure optimization (shifting toward balanced, Japanese, or Greek models) can significantly reduce pressure on cultivated land supply, thereby improving the supply–demand matching stability in most land-use scenarios.

### 3.4. Response of Land-Use Structure to Dietary Structure

**Figure 11:**
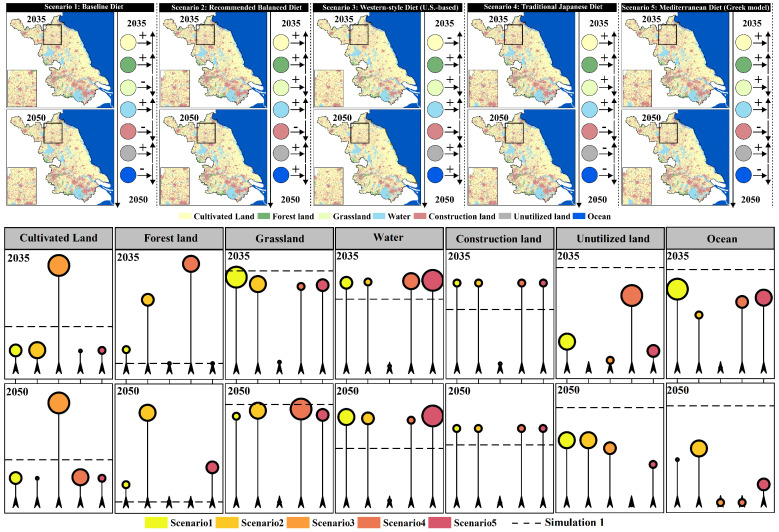
Impact of Different Dietary Structures on Land-Use Structure in the Natural Development Scenario.

In the natural development scenario (Simulation 1), when different dietary structure scenarios (Scenario 1–5) are incorporated as demand-side constraints, the land-use patterns show significant land type sensitivity in response to “dietary structure differences” (Figure 11). In terms of cultivated land, overall, between 2035 and 2050, the cultivated land area in Scenario 3 (U.S. dietary structure) decreases, while the cultivated land areas in Scenario 1, 2, 4, and 5 increase. Compared to Simulation 1, Scenario 3 shows a much higher cultivated land area than the baseline scenario, while Scenario 1, 2, 4, and 5 result in lower cultivated land areas than the baseline scenario. This suggests that, after incorporating different dietary structure scenarios, the higher the cultivated land demand intensity (as in Scenario 3), the more significant the “compensatory adjustment” demand on land-use structure to supply cultivated land, leading to a noticeable increase in cultivated land area compared to the baseline scenario (Simulation 1). In contrast, Scenario 1 (current), Scenario 2 (balanced), Scenario 4 (Japanese), and Scenario 5 (Greek) exert weaker pressure on cultivated land demand, with cultivated land areas being closer to or lower than the baseline natural development scenario (Simulation 1). In terms of forest land, overall, between 2035 and 2050, the forest area increases in all five scenarios (Scenario 1–5). Compared to Simulation 1, the forest area increases significantly when incorporating the dietary structure demands of Scenario 1, 2, 4, and 5, far surpassing the baseline scenario, while Scenario 3 remains nearly the same as the baseline. This indicates that the higher the cultivated land demand intensity, the less pronounced the structural change in forest land. Conversely, the lower the cultivated land demand intensity, the more noticeable the response in forest land change, leading to a significant increase in forest area compared to the baseline scenario (Simulation 1), particularly in Scenario 2 and Scenario 4. For grassland, overall, between 2035 and 2050, the grassland area increases in Scenario 2–5, while Scenario 1 shows a decrease in grassland area. Compared to Simulation 1, when incorporating the dietary structure demands of Scenario 1, 2, 4, and 5, the grassland area is slightly lower than in the baseline scenario, while Scenario 3 shows a much lower grassland area than the baseline scenario. This suggests a negative correlation between cultivated land demand intensity and grassland area. The higher the cultivated land demand intensity, the more significant the response in grassland area, leading to a noticeable decrease in grassland area compared to the baseline scenario (Simulation 1). Changes in water bodies, unused land, and ocean also show noticeable differentiation based on dietary structure scenarios. Water bodies exhibit a larger area than the baseline scenario when incorporating Scenario 1–5 dietary structure demands. Unused land and ocean show a smaller area than the baseline scenario when incorporating Scenario 1–5 dietary structure demands. Notably, the changes in unused land and ocean become more pronounced over time. However, the changes in construction land are minimal across different dietary structure scenarios, indicating that the expansion of construction land in the baseline scenario (Simulation 1) does not show a significant response to dietary structure changes. Instead, dietary structure impacts land-use structure mainly by altering the “cultivated land demand-ecological/unused land regulation” relationship. Overall, under the Simulation 1 scenario, high-resource-consuming diets (especially the U.S. dietary structure) significantly amplify the pressure on cultivated land protection and squeeze adjustable ecological space, while more moderate dietary structures like those in the balanced, Japanese, and Greek scenarios can reduce cultivated land demand and mitigate the intensity of ecological land type conversions, all while not altering the primary trend of urban expansion.

**Figure 12:**
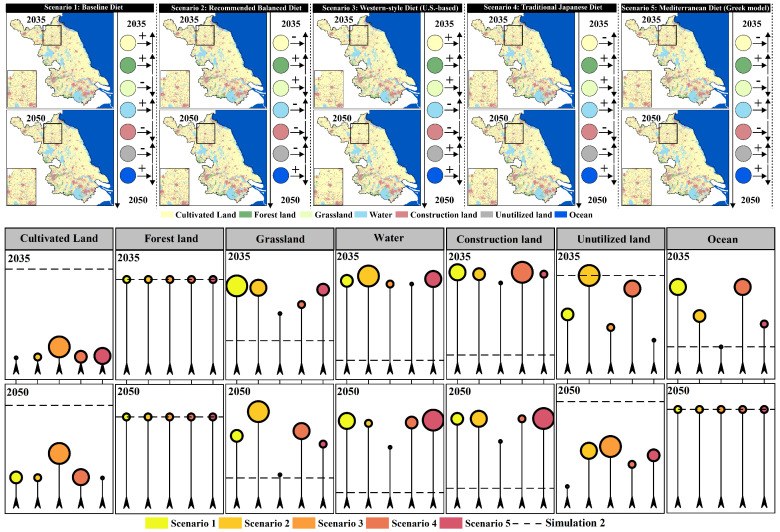
Impact of Different Dietary Structures on Land-Use Structure in the Cultivated Land Protection Scenario.

In the cultivated land protection scenario (Simulation 2), when different dietary structure scenarios (Scenario 1–5) are incorporated as demand-side constraints, the response of land-use patterns to “dietary structure differences” still exhibits significant land type sensitivity. However, compared to the natural development scenario, its effects are more characterized by structural redistribution under the constraints of the cultivated land protection red line rather than unconstrained expansion (Figure 12). In terms of cultivated land, under the rigid constraint of the cultivated land protection goal, the overall fluctuations in cultivated land area across different dietary scenarios are significantly compressed. Compared to Simulation 2, the cultivated land area is generally lower when dietary constraints are incorporated. Scenario 3, with the highest demand intensity, consistently shows the highest cultivated land area in both time periods, and its values are closest to the baseline. Scenario 1 and Scenario 3 show an increase between 2035 and 2050, while Scenario 2, Scenario 4, and Scenario 5 show a more noticeable overall decrease, with Scenario 2 showing the smallest variation. This indicates that under the cultivated land protection framework, “high-demand diets” primarily manifest as an effort to maintain higher cultivated land occupancy rather than further exceeding the red line. For forest land, Scenario 1–5 in 2035 and 2050 are nearly identical to the baseline scenario, with a consistent slight increase in both periods, indicating that in the cultivated land protection scenario, the disturbance to forest land from dietary structure changes is minimal, and the structural stability of forest land is the highest. For grassland, the response caused by dietary differences is more pronounced. Overall, most scenarios show higher levels of grassland than the baseline, with Scenario 2, Scenario 3, and Scenario 4 showing the most significant increases in grassland between 2035 and 2050. Scenario 1 and Scenario 5 show a decrease, and Scenario 3 remains at a relatively low level, closer to the baseline. In terms of water bodies and construction land, after incorporating dietary scenarios, both land types are generally higher than the baseline, but the temporal evolution differs significantly. Water bodies show the strongest increase in Scenario 2 by 2035, but by 2050, Scenario 5 is the most prominent. Construction land responds most strongly to Scenario 4 by 2035, while by 2050, Scenario 5 shows a significant increase. For unused land and ocean, after incorporating dietary constraints, unused land is generally lower than the baseline by 2050, but differences between scenarios remain evident, with Scenario 3 and Scenario 2 being relatively higher. Ocean shows some scenario differentiation in 2035, but by 2050, mostly aligns with the baseline, indicating that adjustments mainly occur in the short term and tend to converge after the constraints are applied. Overall, in the Simulation 2 scenario, the cultivated land protection constraint shifts the impact of dietary structure differences on land-use from “significant increases or decreases in cultivated land” to the “substitution and adjustment of flexible land types, such as grassland, water bodies, and unused land.” High-resource-consuming diets (especially Scenario 3) primarily exert pressure on ecological and flexible spaces by maintaining higher cultivated land occupancy, while relatively moderate or balanced dietary structures (Scenario 2/4/5) are more likely to relieve pressure on cultivated land and enhance structural adjustments in ecological land types and water bodies.

**Figure 13:**
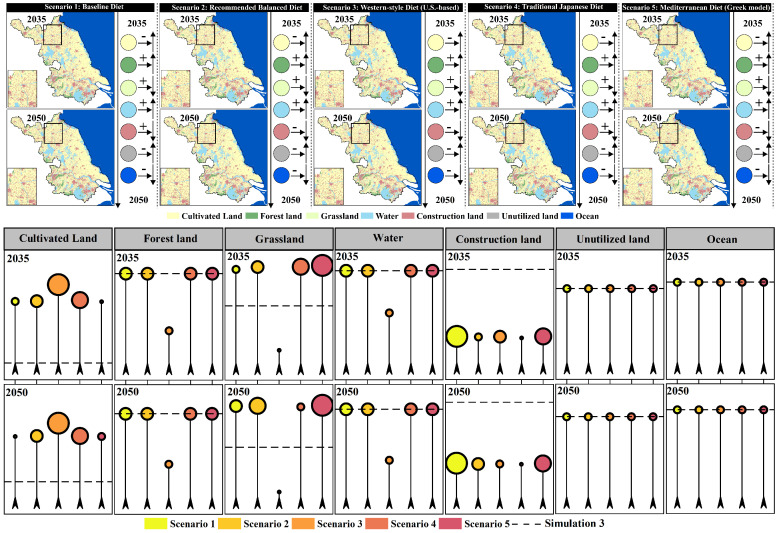
Impact of Different Dietary Structures on Land-Use Structure in the Ecological Protection Scenario.

In the ecological protection scenario (Simulation 3), when different dietary structure scenarios (Scenario 1–5) are incorporated as demand-side constraints, the response of land-use patterns to “dietary structure differences” also shows significant land type sensitivity (Figure 13). However, the core characteristic is that, within the overall framework of ecological priority, the system primarily adapts to different dietary demand pressures through “cultivated land–grassland–forest land–water bodies” structural adjustments. In terms of cultivated land, between 2035 and 2050, the cultivated land area for all dietary scenarios generally shows a declining trend. However, compared to the baseline scenario of Simulation 3, the cultivated land areas in Scenario 1–5 are all significantly higher, with Scenario 3 being the highest and Scenario 4 coming second. This indicates that under ecological protection constraints, when dietary demand intensity rises, land-use structure will prioritize increasing cultivated land occupation to meet the supply needs, thereby creating a stronger compensatory displacement effect on “ecological protection.” For forest land, in Scenario 1, 2, 4, and 5, the forest area is almost identical to the baseline scenario and remains stable (with minimal changes between 2035 and 2050), while in Scenario 3, the forest area is significantly lower than the baseline and remains at a low level in both periods. This suggests that high-resource-consuming diets directly amplify the conversion pressure on forest land, making it a prominent source of structural conflict in the ecological protection scenario. For grassland, after incorporating dietary scenarios, the grassland area is generally higher than the baseline and further increases by 2050, showing clear scenario differentiation. Scenario 5 and Scenario 4 exhibit the most significant increases in grassland area, followed by Scenario 2. Scenario 3 is relatively lower but still higher than the baseline, indicating that grassland plays a stronger “buffer/substitution” role in the ecological protection scenario, helping to reconcile the relationship between cultivated land expansion and ecological protection. For water bodies, Scenario 1, 2, 4, and 5 are essentially consistent with the baseline scenario, while Scenario 3 is significantly lower than the baseline and remains low in both periods. This suggests that, driven by high cultivated land demand, water bodies become another type of flexible space that may be encroached upon or compressed. Meanwhile, construction land remains at a relatively low level across all dietary scenarios, significantly lower than in the baseline scenario. This indicates that under ecological protection constraints, the expansion of construction land is notably dulled, with its expansion efficiency hindered. Dietary structure changes mainly affect land-use structure by “substituting cultivated land with ecological/water bodies and other land types.” Finally, unused land and ocean in Scenario 1–5 are almost identical to the baseline scenario, with minimal changes, reflecting their overall stability in the ecological protection scenario and their insensitivity to dietary structure changes. Overall, under the Simulation 3 scenario, high-resource-consuming diets (especially Scenario 3) lead to a significant increase in cultivated land occupation and compression of forest land and water bodies, intensifying the risk of ecological space displacement. In contrast, more moderate dietary structures (Scenario 2/4/5) cause weaker disturbance to ecological land types, with a greater tendency to achieve structural balance under supply constraints by increasing flexible land types like grassland.

**Figure 14:**
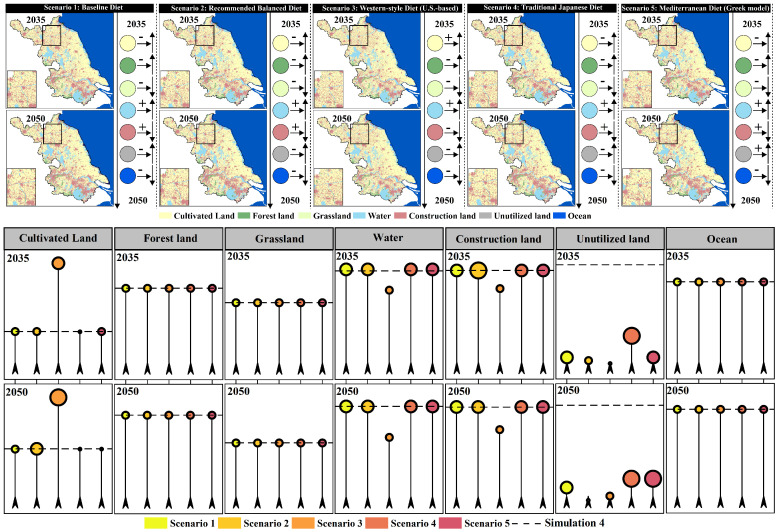
Impact of Different Dietary Structures on Land-Use Structure in the Economic Development Scenario.

In the economic development scenario (Simulation 4), when different dietary structure scenarios (Scenario 1–5) are incorporated as demand-side constraints, the response of land-use patterns to “dietary structure differences” also shows significant land type sensitivity. However, the mechanism of action is more focused on the “rebalancing of cultivated land–construction land–water bodies–unused land” structure under the pressure of construction land expansion (Figure 14). In terms of cultivated land, between 2035 and 2050, the changes in cultivated land across different dietary scenarios show differentiation: Scenario 3 is significantly higher than the baseline scenario in both periods, with the largest increase. This indicates that even in the context of rapid construction land expansion driven by economic development, high-resource-consuming diets still significantly increase the pressure on cultivated land protection. In contrast, Scenario 1, Scenario 2, Scenario 4, and Scenario 5 show cultivated land levels closer to or slightly lower than the baseline, indicating that their cultivated land demand pressure is relatively manageable. For construction land, the response to dietary differences is generally not sensitive, but there is a significant exception: Scenario 3 is notably lower than the baseline economic development scenario in both 2035 and 2050, while the other scenarios (Scenario 1, 2, 4, and 5) are mostly consistent with the baseline. This suggests that under an economic development-oriented scenario, when dietary demand pushes cultivated land to higher levels, construction land expansion is mainly constrained (or its growth is delayed) to release space for cultivated land supply, creating a strong competitive “cultivated land expansion–construction land suppression” relationship. For forest land and grassland, the values in all dietary scenarios are mostly close to the baseline in both time periods, with small differences, indicating that in the economic development scenario, the overall pattern of ecological land types is more stable, and dietary structure has limited direct disturbance on these land types. For water bodies, except for Scenario 3, which is significantly lower than the baseline in both periods, the other scenarios are generally consistent with the baseline. This shows that high cultivated land demand not only may squeeze construction space but also creates additional pressure on water bodies. For unused land, all dietary scenarios show significantly lower areas than the baseline in both 2035 and 2050. This indicates that under the economic development context, when dietary constraints are applied, the system tends to utilize unused land as a backup resource to meet the dual demand for cultivated and construction land. Scenario 4 and Scenario 5 retain more unused land, while Scenario 3 shows the lowest level and a higher rate of conversion. Finally, for ocean, the areas across different dietary scenarios and between the two time periods are almost identical to the baseline, with minimal changes. Overall, under the Simulation 4 scenario, the impact of dietary structure differences on land-use primarily propagates by either strengthening or alleviating “cultivated land demand pressure.” Scenario 3 is the most likely to trigger cultivated land expansion, squeeze construction land and water bodies, and accelerate the conversion of unused land. In contrast, more moderate dietary structures, such as Scenario 1, 2, and 5, result in land-use structures that are closer to the baseline economic development path, with smaller adjustments to land types.

### 3.5. Response of Land-Use Conversion to Dietary Structure

**Figure 15:**
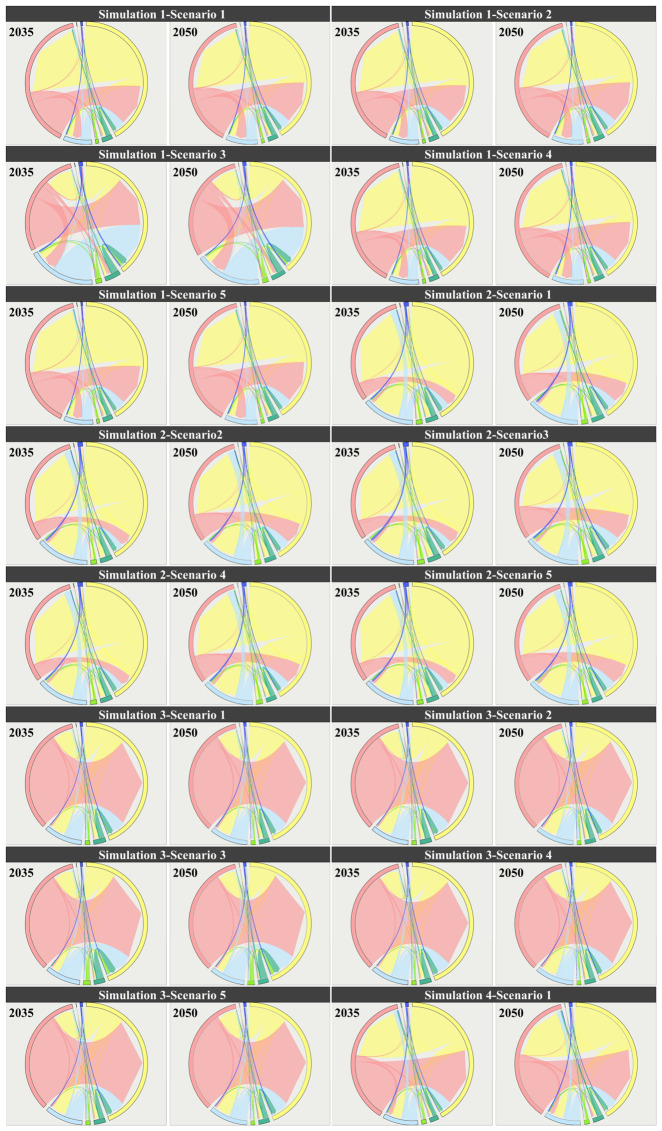

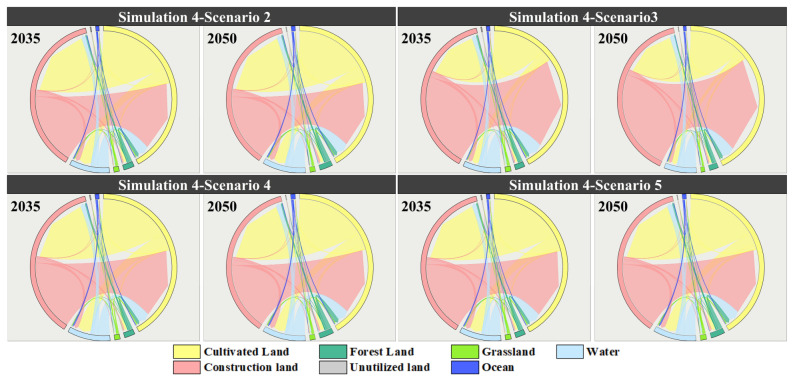
Impact of Different Dietary Structures on Land Type Conversions under Various Development Scenarios.

The results indicate that after incorporating different dietary structure scenarios (Scenario 1–5), land-use transformations are primarily centered around the mutual conversion of “cultivated land–construction land–water bodies” (Figure 15). Among these, cultivated land and construction land are the main land types in the conversion process, followed by water bodies. In the mutual conversion structure centered around cultivated land and construction land, the conversion of water bodies to cultivated land remains a stable and prominent path across all dietary scenarios. This conversion constitutes one of the primary sources for supplementing cultivated land and helps alleviate the pressure of cultivated land conversion to construction land, thus meeting the cultivated land demand required by different dietary structure scenarios. Apart from the major conversion paths mentioned, the conversion of forest land and grassland is relatively small, but under different dietary constraints, they still exhibit some degree of marginal adjustment, mainly converting to cultivated land or construction land. The conversion of unused land and ocean is minimal, typically representing localized replacement and supplementation. It is noteworthy that after incorporating different dietary structure scenarios, dietary structure has a significant impact on land-use structure changes. This is especially evident in the economic development scenario (Simulation 4), which primarily focuses on economic and urban development. In terms of land-use structure, this scenario mainly manifests as large-scale aggregation and expansion of construction land. However, under the influence of dietary structure demand, there is still a considerable amount of construction land converting to cultivated land to maintain a higher level of cultivated land supply to meet the demand of different dietary structures.

Specifically, in the natural development scenario (Simulation 1), after incorporating different dietary structures (Scenario 1–5), the interaction between cultivated land and construction land occurs. The main conversion is from cultivated land to construction land, followed by the conversion of construction land to cultivated land and water bodies to cultivated land. However, these conversions exhibit relatively stable structural characteristics across the different dietary structures. Looking at the impact of different dietary structures, the land-use conversion patterns of Scenario 1, 2, 4, and 5 are quite similar, primarily characterized by the bidirectional conversion between cultivated land and construction land, as well as the conversion between water bodies and cultivated land. Scenario 3 (U.S. dietary structure) significantly differs from these scenarios, with a larger conversion of water bodies and construction land to cultivated land, surpassing the conversion of cultivated land to construction land. Cultivated land, as one of the most important sources of conversion, mainly shifts to construction land and water bodies, indicating that in the context of natural development (Simulation 1), dietary structure impacts are primarily reflected in two core conversion paths: cultivated land to construction land and water bodies to cultivated land. Meanwhile, the conversion of water bodies to cultivated land remains a stable path across all dietary scenarios, with a small amount of conversion to construction land. Construction land also plays an important role in the conversion process, mainly shifting to cultivated land and water bodies, indicating ongoing bidirectional conversion between construction land, cultivated land, and water bodies, rather than one-way expansion. In contrast, the conversion of forest land and grassland is characterized by multiple pathways and small-scale adjustments.

In the cultivated land protection scenario (Simulation 2), after incorporating different dietary structures (Scenario 1–5), the mutual transformation of land-use types between 2035 and 2050 presents a relatively stable conversion structure. The focus of the baseline scenario is on cultivated land protection. However, even with the incorporation of dietary structure influences, a large amount of cultivated land is still converted to construction land and water bodies, leading the land redistribution process. Compared to Simulation 1, the proportion of conversion from construction land and water bodies significantly decreases, with overall conversion intensity weaker than that of cultivated land. The conversion scale of forest land, grassland, unused land, and ocean is similar to that in Simulation 1, with small conversion areas, reflecting more marginal supplementation and localized replacement. Regarding the supplementary paths for cultivated land (to meet the demand for cultivated land), the conversion from water bodies to cultivated land is a stable path, indicating that under cultivated land protection constraints, the replenishment of cultivated land is partially dependent on the conversion of water bodies to cultivated land. Meanwhile, there is continuous bidirectional conversion between cultivated land and construction land, indicating that the coexistence of cultivated land and construction land is an important mechanism for land structure reorganization. Over time, from 2035 to 2050, the overall direction of conversion remains stable, but the proportion of conversion from construction land and water bodies to non-cultivated land types increases, indicating that in the long-term development process, more reliance is placed on the conversion and redistribution of construction land and water bodies to maintain the cultivated land protection goals.

In the ecological protection scenario (Simulation 3), after incorporating different dietary structures (Scenario 1–5), the conversion processes across the five dietary structure scenarios exhibit a high degree of consistency, with no major differences between the scenarios. The baseline scenario focuses on ecological protection, and after incorporating different dietary structures, construction land becomes the primary source of conversion, followed by cultivated land and water bodies. Under the strong constraints of ecological protection, the high demand for cultivated land is mainly supplemented by the conversion of construction land and water bodies. In contrast, forest land and grassland exhibit more small-scale exchanges and compensatory conversions between cultivated land, water bodies, and forest/grassland. The conversion of unused land and ocean remains small and contributes limitedly to the overall pattern.

In the economic development scenario (Simulation 4), after incorporating different dietary structures (Scenario 1–5), compared to the baseline scenario, changes in dietary structure further lead to a strongly coupled pattern of “cultivated land–construction land–water bodies.” In this baseline scenario, economic development is the main driver, with further large-scale expansion of construction land. However, after incorporating different dietary structure influences, a significant amount of construction land is converted to cultivated land, becoming the primary source of cultivated land supply to meet the high demand caused by dietary structure changes. Construction land plays a central role in the expansion of urban areas and the adjustment of the cultivated land supply–demand pattern. Similarly, the conversion from cultivated land to water bodies also holds a relatively high proportion, with water bodies primarily converting to cultivated land, and cultivated land mainly converting to construction land, reflecting a clear bidirectional displacement and structural redistribution between construction land and cultivated land. In contrast, the conversion of forest land and grassland is significantly smaller, mainly shifting to cultivated land and water bodies, serving more as supplementary ecological adjustments, with the conversion of unused land and ocean contributing minimally to the overall pattern. Regarding the impact of different dietary structures, the conversion structures of Scenario 1, 2, 4, and 5 are similar, while in Scenario 3, construction land and water bodies shift more intensively to cultivated land, indicating that this dietary structure places more pressure on the adjustment of cultivated land patterns and further amplifies the dominant conversion relationships between construction land, water bodies, and cultivated land.

**Figure 16:**
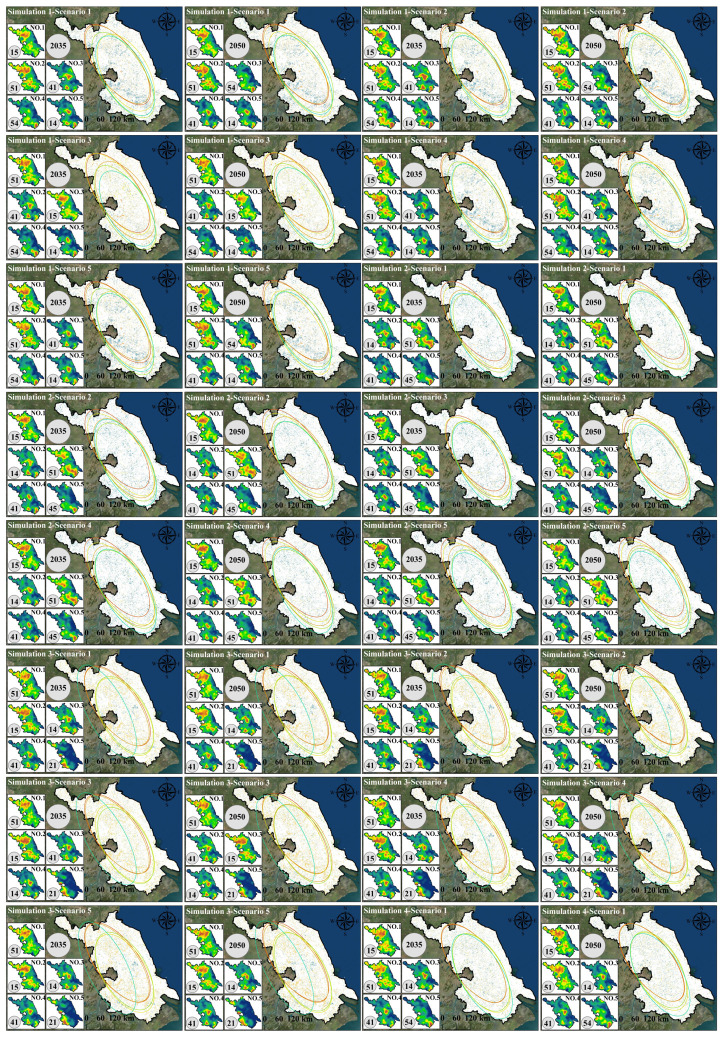

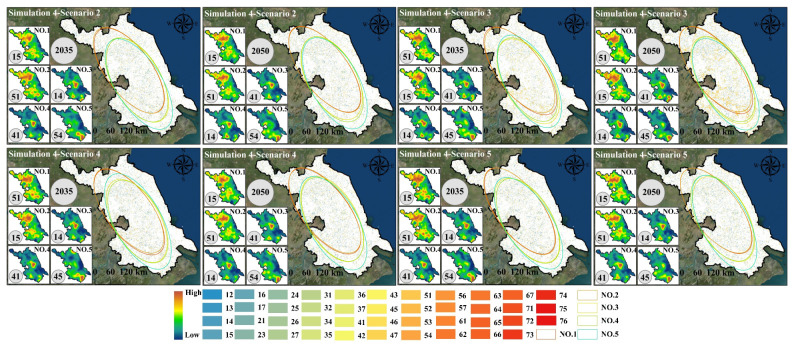
Spatial Characteristics of Prominent Land Type Conversion Paths under Different Dietary Structures in Each Development Scenario. Land-use type codes are defined as follows: 1 indicates cultivated land, 2 forest land, 3 grassland, 4 water bodies, 5 construction land, 6 unused land, and 7 ocean. For the two-digit land-use conversion codes, the first digit indicates the land-use type converted from, and the second digit indicates the land-use type converted to. For example, 12 indicates cultivated land converted to forest land, and 21 indicates forest land converted to cultivated land. By the same rule, 13, 14, 15, 16, and 17 indicate cultivated land converted to grassland, water bodies, construction land, unused land, and ocean, respectively; 23, 24, 25, 26, and 27 indicate forest land converted to grassland, water bodies, construction land, unused land, and ocean, respectively; 31, 32, 34, 35, 36, and 37 indicate grassland converted to cultivated land, forest land, water bodies, construction land, unused land, and ocean, respectively; 41, 42, 43, 45, 46, and 47 indicate water bodies converted to cultivated land, forest land, grassland, construction land, unused land, and ocean, respectively; 51, 52, 53, 54, 56, and 57 indicate construction land converted to cultivated land, forest land, grassland, water bodies, unused land, and ocean, respectively; 61, 62, 63, 64, 65, and 67 indicate unused land converted to cultivated land, forest land, grassland, water bodies, construction land, and ocean, respectively; and 71, 72, 73, 74, 75, and 76 indicate ocean converted to cultivated land, forest land, grassland, water bodies, construction land, and unused land, respectively.

Further, from a spatial perspective, after incorporating different dietary structure scenarios (Scenario 1–5), the land-use conversion patches under each development scenario (Simulation 1–4) exhibit significant northwest–southeast strip-like aggregation patterns. The hotspots for different conversion types are generally distributed along the “Northern Jiangsu–Central Jiangsu–Southern Jiangsu” axis, with the standard deviation ellipse direction consistent and overlapping, indicating a clear path dependence in the spatial organization of the conversion process (Figure 16). Looking further, the hotspots of different conversion patch types are unevenly distributed, showing a pattern of continuous high values in central Jiangsu and more patchy enhancement in the southern coastal areas. The northern and central parts of Jiangsu are more prone to forming continuous aggregations that extend toward the south, while the southern and coastal areas often exhibit secondary hotspot patches, reflecting localized clustering features in the conversion activities of these regions. In terms of temporal evolution, from 2035 to 2050, there is an overall trend of stable aggregation positions with slight adjustments in the range of aggregation. In most scenarios, the direction does not change significantly, indicating that hotspots do not experience systematic migration. The changes mainly manifest as adjustments in the degree of expansion and contraction and the internal connectivity of hotspots. Over the long term, this is more about the continuous expansion or local outward movement of existing hotspots rather than the creation of new independent aggregation cores. Different development scenarios have a moderating effect on this change characteristic. Specifically, in the ecological protection scenario (Simulation 3), hotspots are more concentrated, with weaker outward expansion, reflecting the suppression of the extension and expansion of ecological land type conversions by ecological space constraints. Similarly, in the cultivated land protection scenario (Simulation 2), there is high overlap and stable hotspots, with stronger spatial convergence overall. In contrast, in the natural development (Simulation 1) and economic development (Simulation 4) scenarios, hotspots are concentrated, with more noticeable adjustments in the hotspots under the economic development scenario. After incorporating different dietary structures, the main spatial change is not a systematic shift in hotspot location, but a change in aggregation intensity within largely stable hotspot areas. The standard deviation ellipses in each dietary scenario generally overlap, indicating that dietary differences rarely alter the spatial location of conversion aggregations. The main effect is reflected in changes in hotspot strength and contiguity within different sub-regions (Northern Jiangsu, Central Jiangsu, Southern Jiangsu, and coastal areas). For example, the U.S. dietary structure scenario typically makes conversion patch hotspots more continuous, with the southern extension being more contiguous. In contrast, the balanced dietary structure, Japanese, and Greek dietary structure scenarios generally maintain the existing spatial characteristics of hotspots. In summary, the spatial pattern can be summarized as: stable spatial structure → internal intensity adjustment → scenario modulation (ecological and cultivated land protection tend toward convergence, while economic and natural development are more likely to expand outward).

## 4. Discussion

### 4.1. Dietary Structure Upgrading Drives the Structural Shift in Cultivated Land Pressure

Existing research suggests that rapid economic development, rising incomes, and dietary diversification tend to increase the consumption of meat, dairy products, and high-fat foods, thereby intensifying land occupation and ecological pressure [13]. In contrast, more plant-based diets can reduce land demand and environmental burdens [64]. The findings of this study indicate that the effect of dietary upgrading on cultivated land demand is not simply a matter of overall increase or decrease, but a structural shift in the dominant sources of pressure. In Jiangsu, cultivated land demand is gradually shifting from a grain-dominated pattern toward a structure increasingly constrained by meat consumption and associated feed-grain demand. This transition is particularly evident in economically developed regions, where dietary upgrading moves cultivated land pressure beyond staple-food security alone and toward a more complex coupling among feed grains, livestock products, and oil crops, in line with changing consumer preferences for healthier, higher-protein, and higher-quality foods [65]. From this perspective, the present study emphasizes not only how much cultivated land should be protected, but also which components of food demand are driving land pressure and therefore deserve greater policy attention [40]. Unlike the national average or agricultural provinces in the central and western regions, Jiangsu is located within the Yangtze River Economic Belt and the core of the Yangtze River Delta [26]. Jiangsu is densely populated, highly urbanized, and strongly integrated into the industrial agglomeration dynamics of the Yangtze River Delta; compared with less-developed regions, it also tends to exhibit higher income and consumption levels, alongside more diversified food preferences and a greater shift toward animal-source foods [66]. However, Jiangsu also faces a spatial pattern where construction land expansion and the rigid constraints of cultivated land protection coexist. In other words, demand upgrading and supply-side land constraints are coupled within the same spatiotemporal unit in Jiangsu, making supply–demand conflicts more pronounced. Most studies suggest that consumption upgrading is currently outpacing local land carrying capacity [67]. Therefore, under the limited cultivated land resources, in the coming decades, if residents’ dietary structure shifts toward the U.S. dietary structure (Scenario 3), the land demand for food consumption will significantly increase. This may place stronger constraints on rapid urban economic development and further intensify pressure on agricultural resources and the ecological environment. In Scenario 3, a large amount of animal-based food is required, which not only requires cultivated land but also other agricultural resources like grasslands. If grassland resources are considered, the actual land demand for animal-based food will be higher than what this study calculates. In fact, the rapid development of animal husbandry in China has already led to issues such as overgrazing, which has caused soil degradation, desertification, and other ecological environmental problems [68]. In recent years, China’s meat-intensive dietary pattern has already placed increasing pressure on agricultural resources and the agro-ecological environment. Moreover, the increased consumption of processed foods like plant oils has further intensified the pressure on agricultural resources [8,69]. In addition to the seven major food categories outlined in this study, tea and coffee are also among the major agricultural products consumed today. China is the world’s largest consumer of tea, and coffee consumption is growing at a rate of 15% annually [70]. However, beverages such as coffee and tea are cultural consumption needs, not necessarily for energy supply, and these foods typically have higher land demands. Research shows that as China’s residents’ income levels rise and the middle class expands, meat consumption tends to stabilize while the consumption of stimulants, including coffee, will gradually increase [71]. Therefore, if further consideration is given to these stimulant food categories, cultivated land resources will face even greater pressure.

### 4.2. High-Demand Dietary Scenarios Can Constrain Urban Development and Construction Land Expansion

High-demand dietary scenarios do not merely increase pressure on cultivated land; they may also reshape urban expansion pathways and land-use structure [72]. Existing studies often treat land supply as a passive response to food demand or absorb demand gaps through macro-level trade and market adjustment [73]. By coupling different dietary structure scenarios with alternative land-use development scenarios, the present study identifies a clearer feedback mechanism within the regional land system. When dietary scenarios push cultivated land demand to a high level, especially under resource-intensive diets such as Scenario 3, the land system responds not only through compensatory conversion among flexible land types, such as water bodies and unused land, but may also experience stronger competition between cultivated land and construction land [74]. In this sense, high food-demand pressure can constrain or delay construction land expansion, even under an economically development-oriented scenario. This finding suggests that food and cultivated land security may impose reverse constraints on urban expansion when demand-side pressure becomes sufficiently strong [75]. From the supply side, different development goals and land-use constraints further shape the intensity, direction, and form of construction land expansion, thereby influencing the risk of cultivated land shrinkage and fragmentation [76].

Compared with the natural development, ecological protection, and economic development scenarios, the cultivated land protection scenario is more likely to raise the upper limit of cultivated land supply within the same future time frame, thereby providing stronger support for high-demand dietary pathways such as Scenario 3 [77]. Therefore, in economically developed regions such as Jiangsu, supply-side governance should not be framed simply as a choice between development and protection. Rather, it requires stronger constraints on the mode of construction land expansion and a shift from extensive outward growth toward more compact and intensive land-use. Such an approach can reduce cultivated land conversion, enhance the continuity of cultivated land patches, and improve the resilience of the land system under high-demand dietary scenarios. Some regions in China have already introduced construction-land reduction policies [78]. Since the 18th National Congress of the Communist Party of China, slowing economic growth, tightening resource constraints, and structural optimization have placed large cities under the dual pressure of construction land shortage and the need to protect cultivated land and ecological space [79]. Through policy intervention and technological means, low-efficiency industrial land, ecologically risky land [80], and long-idle or voluntarily withdrawn rural residential land can be restored to ecological or agricultural uses [81]. This can help to reduce total construction land while improving the allocation efficiency of land quotas [82].

### 4.3. Land-System Feedback Characteristics in Plain River-Network Regions and Implications for Multi-Objective Collaborative Governance

Under multiple coupled scenarios, the conversion of water bodies to cropland emerged as one of the relatively stable and prominent pathways for supplementing cropland supply, suggesting that plain river-network regions exhibit a distinctive regional response to dietary change, likely associated with practices such as polder consolidation, shoreline engineering, and pond remediation. However, this pathway also implies potential crowding-out risks for aquatic ecosystem services, flood storage and regulation, and habitat connectivity. If high-demand diets are accommodated primarily through cropland expansion or ecological-space encroachment, the long-term ecological costs may ultimately outweigh the short-term gains and undermine system sustainability [83]. Existing studies have shown that, in response to urban development, intensive human activities have altered underlying surface conditions, simplified river-network structure, and disrupted both the natural evolution and ecological balance of river systems, thereby impairing river functions to varying degrees [84]. In particular, urbanization-driven expansion of impervious surfaces, encroachment upon water areas, and degradation of river channels have significantly weakened the storage and regulation capacity of river networks, accelerating surface runoff, shortening concentration time, and increasing urban flood pressure [83]. Studies in the Taihu Basin further indicate a marked decline in the storage and regulation capacity of river systems over recent decades [85]. To keep food demand within environmental carrying boundaries, multiple strategies must be combined, including dietary transition, technological progress, and reductions in food loss and waste, among which dietary optimization is one of the indispensable demand-side measures [86]. At the provincial scale, this study operationalizes that logic by coupling different dietary scenarios with different development scenarios. The feedback of the land system is expressed not primarily as a unidirectional change in total cropland area, but rather as a reallocation among cropland, construction land, and water bodies. In particular, the conversion of water bodies to cropland constitutes a relatively stable compensatory pathway across multiple scenarios, whereas high-demand dietary scenarios mainly increase adjustment pressure by intensifying land conversion within existing hotspots rather than shifting their spatial locations. This suggests that, for highly developed plain river-network regions such as Jiangsu, the core impact of dietary upgrading is not simply the amount of land shortage, but rather how pressure is transmitted into the existing spatial structure through land-use reorganization. Under China’s stringent land-use control regime, including cultivated land protection, permanent basic farmland, and ecological redlines, conversion among land categories is not a process of free substitution but one of limited adjustment within clearly defined institutional boundaries [37]. Accordingly, the compensatory conversion of water bodies and the reduced elasticity of construction-land expansion identified in this study should not be interpreted as evidence that land can be reallocated without limit in practice, but rather as constrained feedbacks through which the land system attempts to maintain supply capacity under rising local cropland pressure.

From the perspective of regional governance, future land management can no longer proceed through sectorally fragmented approaches. Instead, food security, ecological security, urban development, and energy transition need to be coordinated within a shared constraint framework in order to avoid mutual crowding-out under limited land resources. Accordingly, the governance priority should not be the unilateral expansion of any single land category, but the establishment of a clearer spatial hierarchy: high-quality cropland and critical aquatic ecological space should be protected first; urban development should rely more on the optimization of existing built-up land and on compact and intensive land-use; and new energy infrastructure should be preferentially deployed on rooftops, underused built-up land, former industrial sites, and other low-conflict spaces so as to minimize additional occupation of cropland and ecologically critical areas [87]. At the same time, pressure on the land system cannot be alleviated solely through supply-side land expansion, but must also be reduced through dietary optimization, agricultural technological progress, and lower food loss and waste [86]. Taken together, the results indicate that moderate-demand dietary scenarios are more likely to form robust matches with most land-use development pathways, whereas the cultivated land protection scenario constitutes the key upper-bound condition for accommodating high-demand diets. This suggests that, for highly developed plain river-network regions such as Jiangsu, the critical task is not to optimize a single objective in isolation, but to enhance the overall robustness of the food–land system through the combined logic of demand-side optimization, supply-side protection, and spatial coordination.

### 4.4. Scale Dependence and Regional Differentiation of the Diet–Land System

Existing studies generally show that shifts toward more plant-based diets tend to reduce agricultural land demand and broader environmental pressures, whereas dietary patterns characterized by high consumption of animal-source foods and fats are usually associated with higher levels of resource use [11,55]. However, the results of this study suggest that when the analytical scale is narrowed to a regional unit such as Jiangsu—characterized by high population density, rapid urbanization, constrained cropland resources, and a dense river-network landscape—the relationship between dietary structure and land pressure cannot be simply summarized as a direct equivalence between a more plant-based diet and lower land-demand pressure [18,88]. From the comparison of the five scenarios in this study, Scenario 3 indeed exhibits the highest cropland demand pressure, but Scenario 5 also remains consistently at a relatively high level rather than constituting a distinctly low-pressure case. This indicates that under constraints of regional local supply, land pressure is determined not only by the proportion of animal-source foods, but also by the combination of fruits and melons, vegetable oils, and other relatively input-intensive foods, as well as by the carrying structure of the local land system [13,89]. In other words, global-scale conclusions regarding the pressure-reducing effects of dietary optimization need to be further refined at the regional scale into a more specific question: which combinations of food categories, under which resource boundaries, can actually reduce local land pressure, rather than relying on a simple binary distinction between plant-based and animal-based diets [90]. The results of this study—especially the pressure ranking across different dietary scenarios and the shift in cropland demand from a grain-dominated pattern toward one jointly driven by animal-source foods and higher-value food categories—clearly reflect this scale-dependent differentiation.

Further, existing coupled studies have shown that feedbacks between social behavior and land-use can significantly alter the long-term trajectory of agricultural land demand, indicating that changes in demand do not merely exert a one-way influence on land-use, but can also reshape the internal allocation logic of land systems through feedback mechanisms [91]. Building on this insight, the present study identifies a more specific regional-scale mechanism: in highly developed plain river-network regions such as Jiangsu, once dietary pressure is transmitted to the land system, the response is expressed less as a simple expansion or contraction of total cropland area and more as a reallocation among cropland, construction land, and water bodies or other relatively elastic land categories. Our results further show that high-demand dietary scenarios do not alter the spatial location of land-conversion hotspots; rather, they intensify the degree of competition and spatial contiguity within existing hotspots. At the same time, the conversion of water bodies to cropland constitutes a relatively stable supplementary pathway across multiple scenarios. This suggests that, at the regional scale, differences in dietary structure do not simply generate a generalized condition of cropland scarcity; instead, they trigger a reordering and compression among different functional land spaces within the land system.

Moreover, existing studies caution against assuming that healthy or sustainable diets will automatically translate into improvements in local supply–demand balance [32,92]. In response, recent research at national and interprovincial scales has begun to show that land pressure is not fully locked within administrative boundaries in practice, but is instead redistributed through interprovincial trade, virtual land transfers, and external market adjustment. In the case of China, the latest evidence indicates that interprovincial trade and changes in final demand play an important role in shaping land-transfer patterns, and that coastal developed regions are increasingly dependent on extra-local land resources to satisfy local consumption [93]. However, in recent years, natural disasters, public health emergencies, and trade conflicts have generated substantial shocks to telecoupled food systems, exposing practical vulnerabilities such as insufficient local supply capacity, high external dependence, and fragile cross-regional coordination. This suggests that trade and extra-local supply should not be over-relied upon [9,94]. Against this background, the present study does not attempt to produce a realistic equilibrium forecast under open-market conditions. Instead, it deliberately confines the analysis to a provincial land-constraint framework, with the aim of identifying the upper bound of local cropland pressure, the risks of supply–demand mismatch, and the direction of land-system feedback implied by different dietary structures. In other words, this study seeks to reveal, from the perspective of local regional supply and territorial resource constraints alone, the kinds of pressure boundaries to which different dietary structures may push the land system, and how such pressure is internalized through land reallocation.

In this sense, the influence of regional dietary restructuring on the land system is first and foremost a question of boundaries, and only secondarily a question of aggregate quantity. In other words, dietary pathways that are often regarded as sustainable on average at broader scales must still be re-evaluated, once placed within a specific region, against local cropland supply, aquatic ecological constraints, the intensity of construction-land expansion, and the feedback mechanisms of the land system itself. This does not negate the pressure-reducing potential of dietary optimization; rather, it indicates that such potential is highly conditional at the regional scale, and that its actual effects depend on local resource endowments, the elasticity of the land system, and the constraints imposed by development objectives. For highly developed plain river-network regions such as Jiangsu, the key issue is therefore not to discuss in abstract terms which dietary pattern can maintain a higher degree of supply–demand matching robustness within given land boundaries, but to identify which dietary structure can maintain a higher degree of supply–demand matching robustness within given land boundaries, and which land categories ultimately bear and absorb the pressure generated by high-demand diets.

### 4.5. Limitations and Future Prospects

It should be noted that this study primarily focuses on changes in cropland demand associated with differences in dietary structure and the corresponding land-use responses. Accordingly, several external factors were moderately simplified in the modeling framework. First, with regard to technological progress, the effects of incremental agricultural technological improvement on cropland demand were reflected to some extent through historical yield series and related forecasting models. However, no separate scenarios were constructed for technological leaps, such as breakthrough breeding, substantial improvements in irrigation efficiency, or major upgrades in mechanization. Second, with regard to trade and circulation, this study takes Jiangsu Province as the unit for supply–demand matching and focuses on the relationship between local dietary demand and regional cropland supply. Interprovincial trade, international imports, and logistics-based redistribution networks were not explicitly incorporated into the analytical framework. The main purpose is to identify local supply pressure and its carrying boundary at the regional scale, rather than to treat the results as the absolute supply–demand gap under open circulation conditions. Third, with regard to climate factors, this study does not independently construct scenarios for climate change or extreme weather shocks. Risks such as extreme rainfall, flooding, and high temperatures may further affect supply–demand matching by influencing crop yields, water availability, and the stability of land supply.

Overall, these factors may affect the absolute magnitude of cropland demand and supply–demand matching, but they do not alter the comparative conclusions of this study regarding the relative differences among dietary structures and the direction of land-system responses. In addition, constrained by data availability and research scale, this study selects Jiangsu Province, a representative region characterized by rapid economic development and pronounced land resource constraints, to examine supply–demand balance and response characteristics under the multi-scenario coupling of dietary structure and land-use structure. The analysis has not yet been extended to interprovincial or larger spatial scales. In fact, under the current context of highly developed national and global food trade, supply–demand matching at larger scales is often shaped jointly by cross-regional adjustment, market circulation, and logistics systems. On this basis, future research could build on the present framework by further incorporating interprovincial supply mechanisms, modern food circulation systems, and climate change and extreme weather factors, so as to provide a more comprehensive understanding of the dynamic evolution of food–land systems in the context of sustainable consumption transition.

## 5. Conclusions

This study takes Jiangsu Province, a representative rapidly developing economic region in China, as a typical case and systematically examines the relationships among demand-side dietary transition, supply-side land-use constraints, and land-system feedback responses within a coupled framework of dietary structure scenarios and land-use development scenarios. The results show that the core contradiction in the supply and demand of cultivated land-use in Jiangsu lies not simply in the reduction of cultivated land per se, but in the increasing mismatch between the structure of cropland demand and the structure of land supply under the combined effects of dietary upgrading, urban expansion, and ecological constraints.

(1) From the demand-side perspective, the food consumption of Jiangsu residents from 1990 to 2023 has clearly shifted from a traditional grain-centered pattern toward a more diversified consumption structure. Accordingly, the main source of cropland demand pressure has gradually changed from being grain-dominated to being jointly driven by animal-source foods and high-value food products. Land demand differs significantly across the five dietary scenarios, and such differences are not the result of synchronous changes across all food categories; rather, they are mainly shaped by key food types such as grain, livestock and poultry meat, vegetable oil, and fruits and melons. Overall, more resource-intensive diets correspond to higher levels of land demand, whereas more moderate and balanced dietary structures are associated with lower cropland pressure. This suggests that the impact of dietary restructuring on cultivated land-use is essentially reflected in differences in resource occupation caused by the reconfiguration of food composition, rather than merely by changes in the total amount of consumption.
(2) From the supply-side perspective, the carrying capacity of different land-use development scenarios for dietary demand is not the same. All four land-use scenarios for 2035 and 2050 show a common pattern of construction land expansion under constrained cropland resources. Among them, however, the cultivated land protection scenario is able to provide relatively higher and more stable cropland supply in both periods, and therefore offers the strongest support for high-demand dietary structures. Although the natural development, ecological protection, and economic development scenarios are generally capable of accommodating medium-demand diets, their capacity to support highly resource-intensive diets is clearly insufficient. In particular, under the economic development scenario, the decline in cropland supply by 2050 further widens the supply–demand gap. These findings indicate that in highly developed regions, development pathways that rely solely on the natural evolution of the land system or are oriented primarily toward urban expansion are unlikely to provide stable support for high-demand diets. Cultivated land protection therefore remains the key constraint for enhancing the robustness of regional food systems.
(3) From the feedback-response perspective, once dietary scenarios are incorporated into land-use simulation as demand-side constraints, the land system does not passively accept demand change; instead, it responds through structural adjustment via the reallocation among different land categories. In general, high-demand diets significantly intensify pressure for cropland protection and are more likely to induce compensatory adjustment by compressing more elastic land types such as grassland, woodland, water bodies, and unused land. Under different development objectives, these responses also show strong scenario dependence. Under the natural development scenario, the response is mainly reflected in cropland expansion and grassland compression. Under the cultivated land protection and ecological protection scenarios, adjustment relies more on substitution among grassland, water bodies, and unused land. Under the economic development scenario, by contrast, the most prominent feature is the competitive reallocation among cropland, construction land, and water bodies. Spatially, land conversion hotspots are generally distributed in a northwest–southeast belt extending from northern Jiangsu to central Jiangsu and then to southern Jiangsu, indicating strong spatial path dependence and locational stability. In other words, differences in dietary structure do not remain confined to the demand side, but are further transmitted, under coupled scenarios, into land-structure reorganization and spatial pattern adjustment.

Therefore, for regions such as Jiangsu that are economically developed, densely populated, and strongly constrained by land resources, the focus of future food security governance should no longer be limited to ensuring sufficient staple grain supply. Instead, it should shift toward an integrated governance logic that jointly advances dietary structure guidance, cultivated land protection constraints, and land-system elastic adjustment. Put differently, relying solely on supply-side expansion is insufficient to fundamentally alleviate the resource pressure generated by dietary upgrading. Rather, coordination between demand-side dietary optimization and supply-side cultivated land protection is the key to improving the robustness of regional cropland supply–demand matching and enhancing the resilience of the land system.

## Figures and Tables

**Table 1:** Conversion rate of original crop to corresponding food.

Year	Grains	Vegetables	Fruits	Oils	Aquatic Products	Eggs	Meats
2011–2023	0.897	0.200	0.100	0.384	1.000	1.000	1.000
2001–2010	0.758	0.200	0.100	0.306	1.000	1.000	1.000
1990–2000	0.596	0.200	0.100	0.210	1.000	1.000	1.000

Note: Conversion ratios from raw crops to corresponding food products are taken from the literature [52,53,54].

**Table 2:** Five typical dietary structure scenarios.

Dietary Structure Scenarios	Grains	Vegetables	Fruits	Oils	Aquatic Products	Eggs	Meats
Current dietary structure scenario (Scenario 1)	Calculated based on the observed annual per capita food consumption						
Balanced dietary structure scenario (Scenario 2)	146.00	90.88	33.22	14.60	25.55	20.07	43.8
U.S. dietary structure scenario (Scenario 3)	105.36	118.27	115.00	36.00	22.79	16.12	127.81
Japanese dietary structure scenario (Scenario 4)	129.86	115.95	55.00	13.00	46.20	19.86	53.32
Greek dietary structure scenario (Scenario 5)	112.45	153.38	150.00	22.00	21.72	8.74	72.69

Note: Parameters for the U.S., Japanese, and Greek dietary structure scenarios are calculated from the FAOSTAT database, whereas parameters for China’s balanced dietary structure scenario are based on the Chinese Dietary Guidelines.

**Table 3:** Evaluation Results of LSTM Model Performance and Parameters.

Specific Indicators		Training Set					Test Set				
		R^2^	MAE	MBE	nMAE%	nMBE%	R^2^	MAE	MBE	nMAE%	nMBE%
Consumption of different food categories	Grains	0.777	10.707	−1.107	5.27%	−0.54%	0.600	9.626	−1.595	4.73%	−0.78%
	Vegetables	0.589	5.014	−0.125	4.45%	−0.11%	0.402	7.164	−0.817	6.36%	−0.73%
	Fruits	0.793	0.738	−0.060	2.83%	−0.23%	0.635	6.847	−4.099	26.26%	−15.72%
	Oils	0.933	0.560	0.010	6.30%	0.11%	0.342	1.824	−0.129	20.52%	−1.45%
	Aquatic products	0.984	0.872	0.0240	7.21%	0.20%	0.941	1.409	0.325	11.65%	2.69%
	Eggs	0.999	0.104	0.013	1.23%	0.15%	0.794	2.538	−0.346	30.03%	−4.09%
	Meats	0.998	0.511	0.080	2.07%	0.32%	0.973	2.953	0.011	11.99%	0.04%
Population	Population size	0.999	32,165.879	3736.199	0.04%	0.00%	0.991	174,531.8	54,854.599	0.23%	0.07%
Yield per unit area for different food categories	Grains	0.998	23.604	−3.726	0.40%	−0.06%	0.645	283.041	−22.463	4.79%	−0.38%
	Vegetables	0.999	244.656	−17.463	0.78%	−0.06%	0.943	2261.146	−315.247	7.23%	−1.01%
	Fruits	0.999	174.92687	6.438	0.57%	0.02%	0.999	201.963	8.473	0.66%	0.03%
	Oils	0.999	4.184	0.340	0.22%	0.02%	0.999	4.020	0.402	0.21%	0.02%
	Aquatic products	0.999	69.205	−20.603	1.43%	−0.43%	0.995	272.521	18.921	5.63%	0.39%
	Eggs	0.999	100.642	1.370	0.95%	0.01%	0.961	899.771	−241.778	8.45%	−2.27%
	Meats	0.999	39.500	7.169	0.51%	0.09%	0.911	370.050	120.488	4.74%	1.54%
Demand area for different food categories	Grains	0.998	28,146.037	−7885.625	1.10%	−0.31%	0.986	150,213.081	48,707.008	5.85%	1.90%
	Vegetables	0.988	7743.783	901.896	0.83%	0.10%	0.935	110,437.052	4254.916	11.88%	0.46%
	Fruits	0.999	6385.208	−30.587	1.55%	−0.01%	0.965	56,983.027	5603.460	13.82%	1.36%
	Oils	0.998	6510.119	−685.853	0.82%	−0.09%	0.870	99,519.453	6921.188	12.49%	0.87%
	Aquatic products	0.999	949.224	109.196	0.73%	0.08%	0.892	12,575.493	3311.232	9.72%	2.56%
	Eggs	0.999	1276.567	161.312	1.44%	0.18%	0.964	7640.219	582.816	8.64%	0.66%
	Meats	0.999	9895.524	1698.177	2.03%	0.35%	0.986	53,074.885	−2197.053	10.90%	−0.45%

**Table 4:** Land-Use Probability Settings under Different Simulation.

Land-Use Type	Natural Development (Simulation 1)							Cultivated Land Protection (Simulation 2)							Ecological Protection (Simulation 3)							Economic Development (Simulation 4)						
	A	B	C	D	E	F	G	A	B	C	D	E	F	G	A	B	C	D	E	F	G	A	B	C	D	E	F	G
A	1	1	1	1	1	1	1	1	0	0	0	0	0	0	1	1	1	1	0	0	0	1	1	1	1	1	0	0
B	1	1	1	0	0	0	0	1	1	1	0	0	1	1	0	1	1	0	0	0	0	1	1	1	1	1	0	0
C	1	1	1	1	1	1	1	1	1	1	1	1	1	1	0	1	1	0	0	0	0	1	1	1	1	1	0	0
D	1	0	1	1	1	1	1	1	0	1	1	0	1	1	0	1	1	1	0	0	0	1	0	1	1	1	0	0
E	1	1	1	1	1	1	1	1	1	1	1	1	0	0	1	1	1	1	1	0	0	0	0	0	0	1	0	0
F	1	1	1	1	1	1	1	1	1	1	1	1	1	1	1	1	1	1	0	1	0	1	1	1	1	1	1	0
G	1	1	1	1	1	0	1	1	1	1	1	1	1	1	1	1	1	1	0	0	1	1	1	1	1	1	0	1

Note: A, B, C, D, E, F, and G denote cultivated land, forest land, grassland, water, construction land, unused land, and ocean, respectively; 1 indicates conversion is allowed between land-use types, whereas 0 indicates conversion is not allowed.

**Table 5:** Neighborhood Weight Settings for Land-Use Types under Different Simulations.

Different Simulation	Cultivated Land	Forest	Grassland	Water	Construction Land	Unused Land	Ocean
Natural development (Simulation 1)	0.7	0.3	0.5	0.5	1	0.8	0.01
Cultivated land protection (Simulation 2)	1	0.4	0.5	0.5	0.3	0.8	0.01
Ecological protection (Simulation 3)	0.8	1	1	0.5	0.02	0.8	0.01
Economic development (Simulation 4)	0.1	0.1	0.1	0.2	0.8	0.8	0.01

**Table 6:** Accuracy assessment (Kappa, OA) under different simulations.

Different Simulation	Kappa	OA
Natural development (Simulation 1)	0.832	0.903
Cultivated land protection (Simulation 2)	0.873	0.928
Ecological protection (Simulation 3)	0.826	0.901
Economic development (Simulation 4)	0.879	0.930

**Table 7:** Pettitt test of food consumption by category.

Food Category	First Change Point	*p*-Value	Second Change Point	*p*-Value
Grain	2007	0.000009 ***	2013	0.026506 ***
Vegetables	2008	0.074337	2020	0.230650
Fruits	2012	0.000151 ***	2016	0.078354
Oils	2012	0.000483 ***	2017	0.048516 ***
Aquatic products	2005	0.000009 ***	2014	0.003341 ***
Eggs	2011	0.000203 ***	2016	0.039434 ***
Meats	2004	0.000012 ***	2013	0.002386 ***

Note: *** indicates *p* < 0.001.

**Table 8:** Mann–Kendall test and Sen’s slope of food consumption by category.

Food Category	Entire Period	Sen’s Slope	Phase I	Sen’s Slope	Phase II	Sen’s Slope	Phase III	Sen’s Slope
Grain	1990–2023	−6.1167 ***	1990–2007	−3.3778 ***	2008–2013	−14.7120 *	2014–2023	1.6333 *
Vegetables	1990–2023	−0.5085 **	1990–2008	−0.8535 *	2009–2020	1.7500	2021–2023	1.7500
Fruits	1990–2023	1.0348 ***	1990–2012	−0.1333	2013–2023	1.2000 **	-	-
Oils	1990–2023	0.0894 ***	1990–2012	0.0375	2013–2017	−0.6808 *	2018–2023	−0.1000
Aquatic products	1990–2023	0.5048 ***	1990–2005	0.2568 ***	2006–2014	0.1625	2015–2023	0.8750 ***
Eggs	1990–2023	0.2000 ***	1990–2011	0.0250	2012–2016	0.5675 **	2017–2023	1.1750 ***
Meats	1990–2023	0.9800 ***	1990–2004	0.4691 ***	2005–2013	0.3467 *	2014–2023	1.0500 ***

Note: *** indicates *p* < 0.001; ** indicates *p* < 0.01; * indicates *p* < 0.05.

**Table 9:** Pettitt change-point test for total food-demand land area under five dietary scenarios, 1990–2023.

	First Change Point	*p*-Value	Second Change Point	*p*-Value
Scenario 1	2007	0.000011	2010	0.369453
Scenario 2	2005	0.000011	2011	0.016862
Scenario 3	2005	0.000009	2011	0.016862
Scenario 4	2006	0.000008	2013	0.031458
Scenario 5	2006	0.000008	2013	0.013153

**Table 10:** Mann–Kendall test and Sen’s slope for total food-demand land area under five dietary scenarios, 1990–2023.

	Entire Period	Sen’s Slope	Phase I	Sen’s Slope	Phase II	Sen’s Slope	Phase III	Sen’s Slope
Scenario 1	1990–2023	−137,128.6567 ***	1990–2005	−243,775.7246 ***	2006–2012	−289,062.0214 ***	2013–2023	−14,964.5688
Scenario 2	1990–2023	−138,291.5908 ***	1990–2007	−226,127.0500 ***	2008–2015	−115,189.5697 *	2016–2023	−69,845.9240 *
Scenario 3	1990–2023	−250,843.6074 ***	1990–2007	−432,942.1184 ***	2008–2015	−191,600.9591	2016–2023	−95,624.2113
Scenario 4	1990–2023	−150,416.3424 ***	1990–2006	−255,306.0704 ***	2007–2014	−153,359.2794 ***	2015–2023	−64,812.0254 ***
Scenario 5	1990–2023	−211,295.1683 ***	1990–2006	−348,903.3715 ***	2007–2014	−225,628.3367 ***	2015–2023	−87,840.5270 **

Note: *** indicates *p* < 0.001, ** indicates *p* < 0.01, and * indicates *p* < 0.05.

**Table 11:** Pairwise comparisons of total food-demand land area across the five dietary scenarios, 1990–2023.

Scenario A	Scenario B	Direction	Scenario A	Scenario B	Direction
Scenario 1	Scenario 2	Scenario 1 < Scenario 2 ***	Scenario 2	Scenario 4	Scenario 2 < Scenario 4 ***
Scenario 1	Scenario 3	Scenario 1 < Scenario 3 ***	Scenario 2	Scenario 5	Scenario 2 < Scenario 5 ***
Scenario 1	Scenario 4	Scenario 1 < Scenario 4 ***	Scenario 3	Scenario 4	Scenario 3 > Scenario 4 ***
Scenario 1	Scenario 5	Scenario 1 < Scenario 5 ***	Scenario 3	Scenario 5	Scenario 3 > Scenario 5 ***
Scenario 2	Scenario 3	Scenario 2 < Scenario 3 ***	Scenario 4	Scenario 5	Scenario 4 < Scenario 5 ***

Note: *** indicates *p* < 0.001.

**Table 12:** Pettitt change-point test for food category-specific land demand under five dietary scenarios, 1990–2023.

	Scenario 1		Scenario 2		Scenario 3		Scenario 4		Scenario 5	
	First Change Point	Second Change Point	First Change Point	Second Change Point	First Change Point	Second Change Point	First Change Point	Second Change Point	First Change Point	Second Change Point
Grain	2006 ***	2013 ***	2010 ***	2019	2010 ***	2019	2010 ***	2019	2010 ***	2019
Vegetables	2008 ***	2020	2008 ***	2014	2008 ***	2014	2008 ***	2014	2008 ***	2014
Fruits	2012 ***	2018	2006 ***	2014 ***	2006 ***	2014 ***	2006 ***	2014 ***	2006 ***	2014
oils	2001 ***	2017	2006 ***	2014 ***	2006 ***	2014 ***	2006 ***	2014 ***	2006 ***	2014 ***
Aquatic products	2012 ***	2017 **	2006 ***	2014 ***	2006 ***	2014 ***	2006 ***	2014 ***	2006 ***	2014 ***
Eggs	2014 ***	2018	2005 ***	2009	2005 ***	2009	2005 ***	2009	2005 ***	2009
meats	2009 ***	2016 **	2001 ***	2016 ***	2001 ***	2016 ***	2001 ***	2016 ***	2001 ***	2016 ***

Note: *** indicates *p* < 0.001; ** indicates *p* < 0.01.

**Table 13:** Mann–Kendall test and Sen’s slope for food category-specific land demand under five dietary scenarios, 1990–2023.

		Entire Period	Sen’s Slope	Phase I	Sen’s Slope	Phase II	Sen’s Slope	Phase III	Sen’s Slope
Grain	Scenario 1	1990–2023	−133,801.7901 ***	1990–2006	−178,284.4985 ***	2007–2012	−172,571.8278 **	2013–2023	−7588.9371
	Scenario 2	1990–2023	−40,002.3106 ***	1990–2010	−58,967.7800 ***	2011–2023	−10,235.2734 **	-	-
	Scenario 3	1990–2023	−28,867.4208 ***	1990–2010	−42,553.7349 ***	2011–2023	−7386.2220 ***	-	-
	Scenario 4	1990–2023	−35,580.1373 ***	1990–2010	−52,449.0131 ***	2011–2023	−9103.7850 **	-	-
	Scenario 5	1990–2023	−30,809.9988 ***	1990–2010	−45,417.3073 ***	2011–2023	−7883.2637 ***	-	-
Vegetables	Scenario 1	1990–2023	−17,925.1009 ***	1990–2004	−28,375.7586 ***	2005–2023	−15,846.8327 **	-	-
	Scenario 2	1990–2023	−11,353.7577 ***	1990–2008	−10,469.5183 ***	2009–2023	−7750.1097 ***	-	-
	Scenario 3	1990–2023	−14,774.8134 ***	1990–2008	−13,624.1396 ***	2009–2023	−10,085.3329 ***	-	-
	Scenario 4	1990–2023	−14,484.9887 ***	1990–2008	−13,356.8867 ***	2009–2023	−9887.4977 ***	-	-
	Scenario 5	1990–2023	−19,160.9105 ***	1990–2008	−17,668.6440 ***	2009–2023	−13,079.2962 ***	-	-
Fruits	Scenario 1	1990–2023	4684.3540	1990–2012	−11,811.2192 ***	2013–2023	−1456.7684	-	-
	Scenario 2	1990–2023	−14,492.8775 ***	1990–2006	−18,861.1457 ***	2007–2014	−9447.1163 ***	2015–2023	−14,058.4947 ***
	Scenario 3	1990–2023	−50,178.5612 ***	1990–2006	−65,302.7777 ***	2007–2014	−32,708.6672 ***	2015–2023	−48,674.6017 ***
	Scenario 4	1990–2023	−23,998.4423 ***	1990–2006	−31,231.7632 ***	2007–2014	−15,643.2756 ***	2015–2023	−23,279.1573 ***
	Scenario 5	1990–2023	−65,450.2973 ***	1990–2006	−85,177.5361 ***	2007–2014	−42,663.4789 ***	2015–2023	−63,488.6109 ***
oils	Scenario 1	1990–2023	−15,816.9091 ***	1990–2001	543.0428	2002–2023	−6005.0244	-	-
	Scenario 2	1990–2023	−39,515.2788 ***	1990–2006	−35,487.2861 ***	2007–2014	−41,527.0084	2015–2023	−14,597.2226 ***
	Scenario 3	1990–2023	−97,434.9341 ***	1990–2006	−87,502.8971 ***	2007–2014	−102,395.3633	2015–2023	−35,993.1516 ***
	Scenario 4	1990–2023	−35,184.8373 ***	1990–2006	−31,598.2684 ***	2007–2014	−36,976.1034	2015–2023	−12,997.5270 ***
	Scenario 5	1990–2023	−59,543.5708 ***	1990–2006	−53,473.9927 ***	2007–2014	−62,574.9442	2015–2023	−21,995.8148 ***
Aquatic products	Scenario 1	1990–2023	942.7374 ***	1990–2012	−695.3988	2013–2017	731.5213	2018–2023	2091.9794
	Scenario 2	1990–2023	−10,424.7020 ***	1990–2006	−10,509.4833 ***	2007–2014	−2713.3890 ***	2015–2023	−13,132.6550 ***
	Scenario 3	1990–2023	−9298.5894 ***	1990–2006	−9374.2123 ***	2007–2014	−2420.2793 ***	2015–2023	−11,714.0199 ***
	Scenario 4	1990–2023	−18,850.1461 ***	1990–2006	−19,003.4493 ***	2007–2014	−4906.4021 ***	2015–2023	−23,746.7187 ***
	Scenario 5	1990–2023	−8862.0167 ***	1990–2006	−8934.0891 ***	2007–2014	−2306.6462 ***	2015–2023	−11,164.0418 ***
Eggs	Scenario 1	1990–2023	480.2484	1990–2014	−1864.4626 ***	2015–2023	5573.5617 ***	-	-
	Scenario 2	1990–2023	−4486.4640 ***	1990–2005	−4343.5196 ***	2006–2023	402.9875	-	-
	Scenario 3	1990–2023	−3602.5803 ***	1990–2005	−3487.7976 ***	2006–2023	323.5944	-	-
	Scenario 4	1990–2023	−4438.4147 ***	1990–2005	−4297.0013 ***	2006–2023	398.6715	-	-
	Scenario 5	1990–2023	−1953.2601 ***	1990–2005	−1891.0267 ***	2006–2023	175.4476	-	-
meats	Scenario 1	1990–2023	15,248.3196 ***	1990–2009	−890.9259	2010–2016	42,443.0819 **	2017–2023	66,943.8376
	Scenario 2	1990–2023	−9742.3100 ***	1990–2001	−73,010.2612 ***	2002–2016	−4994.5271	2017–2023	2930.4004
	Scenario 3	1990–2023	−28,428.4166 ***	1990–2001	−213,046.6092 ***	2002–2016	−14,574.2124 ***	2017–2023	8651.0154
	Scenario 4	1990–2023	−11,859.8167 ***	1990–2001	−88,879.1582 ***	2002–2016	−6080.0955	2017–2023	3567.3276
	Scenario 5	1990–2023	−16,168.2310 ***	1990–2001	−121,167.0294 ***	2002–2016	−121,167.0294 ***	2017–2023	−8288.8624

Note: *** indicates *p* < 0.001; ** indicates *p* < 0.01.

## Data Availability

The original contributions presented in this study are included in the article. Further inquiries can be directed to the corresponding authors.
